# Influence of different base materials and thicknesses on the fracture resistance of endocrown: A three-dimensional finite element analysis

**DOI:** 10.1186/s12903-022-02350-8

**Published:** 2022-08-25

**Authors:** Xia Cheng, Xiu-yin Zhang, Wen-hao Qian

**Affiliations:** 1 Shanghai Xuhui District Dental Center, Shanghai, 200032 China; 2 Dept. of Prosthodontics, Shanghai Ninth People’s Hospital Affiliated to Shanghai Jiaotong University, Shanghai, 200011 China

**Keywords:** Endocrown, Base materials, Three-dimensional finite element analysis, Stress

## Abstract

**Introduction:**

To analyze the stress distribution of the all-ceramic endocrown with different base materials and thicknesses using three-dimensional finite element analysis.

**Methods:**

A endodontically treated maxillary premolar was scanned by micro-CT, a three-dimensional finite element model of the endocrown with fluid resin as the base material was divided into control (0 mm), 1 mm, 2 mm, and 3 mm groups according to base thickness. Three kinds of conventional base materials were used and divided into glass ion group (A), fluid resin group (B), and nanocomposite resin group (C), and a three-dimensional finite element model of the endocrown with 1.0 mm thickness of base was established. A static loading with axial and 45° direction was applied to each model, the stress distribution of each part of the endocrown was analyzed under different base materials and thicknesses.

**Results:**

The different thickness of the base layer has an influence on the components of the restoration and the tooth. The stress in the control group was the largest. The stress was the lowest when the thickness of the base layer was 1 mm; The maximum of the equivalent stress, the first, second, and third principal stress in the endocrown, abutment, and alveolar bone, are basically the same with the different base materials. The stress on the base layer increases with the elastic modulus of base materials increases.

**Conclusions:**

The base layer played a force buffering effect on the dental body restored with endocrowns, and the effect was the best at 1 mm; The selection of base material has little influence on the whole, but in order to protect the weak tissues of the cavity bottom, the base material with lower elastic modulus can be used.

## Introduction

The crown restoration is particularly important for the endodontically treated dead teeth, directly affecting the final treatment effect. In recent years, modern dental restoration has also proposed the concept of "minimally invasive," [1] and how to choose the best repair method has become a hot topic for clinicians. In this context, endocrown has become an important way of tooth restoration after being endodontically treated. The use of base materials significantly impacts the fracture of restorations and teeth [2], but few reports on the selection of base materials and base thickness. Based on the design of different base thicknesses (base material: fluid resin) and base materials (i.e., glass ionomer, fluid resin, composite resin), this experiment repaired the endodontically treated maxillary first premolar model restored with endocrowns. Then, the stress distribution of restorations and the residual teeth were analyzed by the three-dimensional finite element method, and the most suitable base thickness and material were selected to provide a theoretical reference for clinical operation.

## Materials and methods

### Main materials and equipment

CBCT (Newtom VG, Italy); CEREC MC XL CAD / CAM system (Sirona, Germany); glass ionomer cement (GC, Japan); flowable resin (Filtek Z-350 mobile nano-resin, 3 M, USA); nanometer resin (Z-350 A3D, 3 M, USA); all-ceramic system (IPS e.max CAD, Yishoujia Company); adhesive (Variolink N, Yishoujia Company); ProTaper root canal preparation instrumentation (Dentsply, USA); AH-Plus root filling paste (Dentsply, USA).

### Finite element model generation

#### Selection and modeling of extracted teeth

One maxillary first premolar that needed to be extracted for orthodontic purposes was collected and used as a modeling object, requiring a normal appearance, complete dentition, and dimensions within the average range of permanent tooth measurements.

The extracted tooth was scanned with Micro-CT at a layer thickness of 0.3 mm, and tomographic images were acquired. Image data were saved in standard medical format (i.e., DICOM). The data were imported into mimics and reconstructed to obtain geometric models of the teeth. The enamel, dentin, and pulp were extracted according to the grayscale values of different tissues and exported to STL format. After the reconstruction by mimics, the obtained model surface has depressions and unevenness, which interferes with the meshing, so the subsequent surface needs to be processed. The model in STL format was imported into Geomagic software for refinement. Specifically, the surface is smoothed by removing the deviations and restoring the missing information using the purge point data and fill functions, respectively. At the same time, the triangular surfaces are merged to create a fitted surface and closed to obtain the outline of the extracted tooth.

**Fig. 1:**
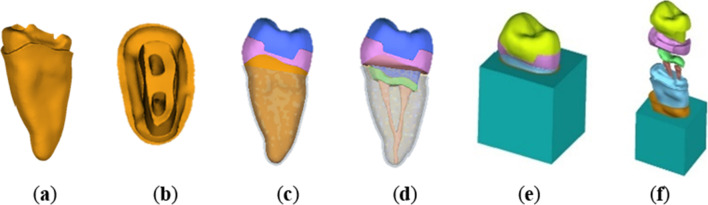
Design of abutment components based on Geomagic software

In Geomagic software, a Boolean operation is performed on the three-dimensional model to complete the drawing design of each component as required, as shown in Fig. 1. Requirements for making the model of endocrown: The axial wall extends along with the proximal and distal mesial directions to the adjacent surface and then towards the cervical part of the tooth, 1.0 mm above the enamel bone boundary, 1.0 mm inward retraction to establish the adjacent surface shoulder, followed by 15° of axial wall abduction to complete the adjacent surface preparation. The functional cusp of the coincidental surface was retracted by 2.0 mm, the non-functional cusp was retracted by 1.0 mm, and the axial surface was uniformly retracted by 1.0 mm to establish the shoulder sill 1.0 mm below the occlusal contact point and at 90° to the long axis of the tooth. The root canal preparation was simulated with a nickel-titanium file (Protaper), the cement filling pattern was drawn, the buccal and lingual root canals were 06 tapered 25#, and the cement was simulated to fill the root canal opening. The thickness of the cement layer was 50 μm, and the modeling was considered as a whole with the restoration because of the complex cement interface and the thin thickness.

In the first group of models, the thickness of the base layer was controlled as 1 mm. In the second group of models, the thickness of the base layer was designed as 0, 1 mm, 2 mm, and 3 mm, respectively.

**Fig. 2:**
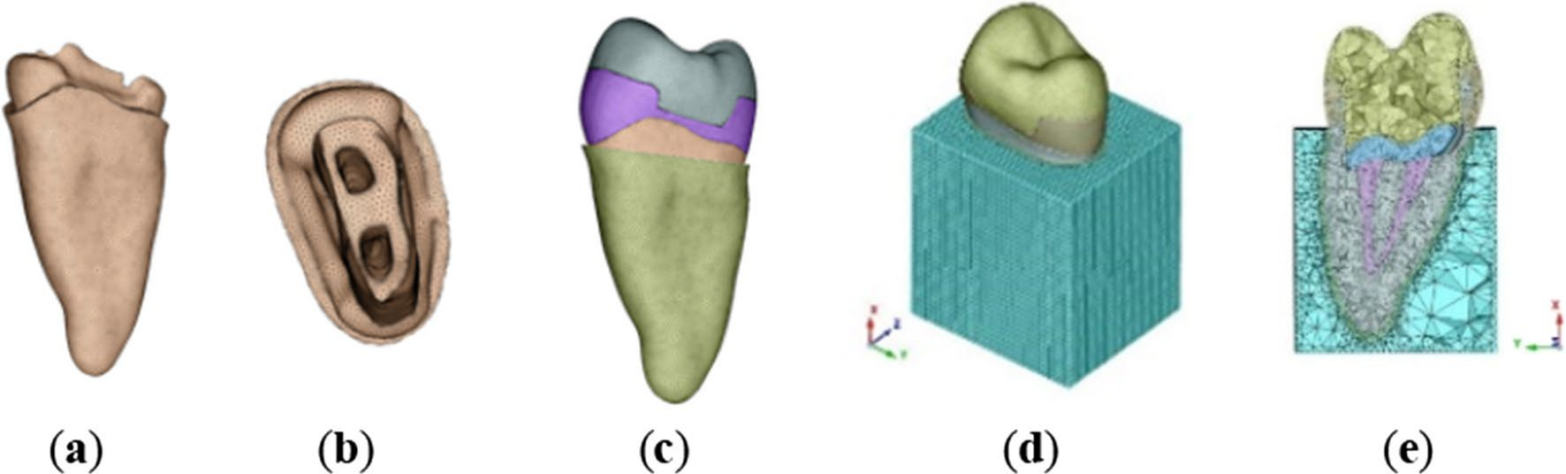
After the design is completed, the model is imported into Hypermesh software for mesh generation

Refine the model and export it as an STL file. The STL file is imported into Hypermesh software for meshing (as shown in Fig. 2), and the fem file is generated. Import the fem file into the FEA software Hyperworks for force analysis and output the results.

#### Experimental groups

1. The established models were divided into four groups according to the thickness of the base material: 0 mm (control group), 1 mm, 2 mm, and 3 mm.
2. The established models were divided into three groups according to the different materials of the base layer: glass ion group (group A), fluid resin group (group B), and nanocomposite resin group (group C).

#### Mesh division and material selection

The alveolar bone model was supposed to be a cube, and the dental model was fixed in it. The tetrahedral meshing method was adopted, with 1 mm sides for the alveolar bone tetrahedra, 0.2 mm for the gutta-percha, and 0.3 mm for the rest of the model. Table 1 shows the number of nodes and units for each part of the model; Table 2 shows the mechanical parameters related to the modeling [3].

**Table 1:** Node number and unit number of each part of the model

Name	Nodes	Unit number	Name	Nodes	Unit number
Enamel	113,858	646,973	Gutta-percha	15,952	83,381
Alveolar bone	85,744	478,802	Periodontal membrane	19,739	76,901
Dentin	33,072	164,258			
Base layer (1 mm)	2556	10,956	Base layer (2 mm)	4824	23,052
Base layer (3 mm)	7131	35,391			
Endocrown (no base layer)	26,126	134,213	Endocrown (base layer 1 mm)	24,273	124,417
Endocrown (base layer 2 mm)	23,131	118,704	Endocrown (base layer 3 mm)	21,306	109,222

**Table 2:** Mechanical parameters related to modeling

Material	Elastic modulus (pa)	Poisson ratio	Material	Elastic modulus (pa)	Poisson ratio
Enamel	8.41E + 10	0.33	Emax all-ceramic endocrown	9.5E + 10	0.24
Dentin	1.86E + 10	0.31	Glassion	9.8E + 09	0.3
Alveolar bone	1.37E + 10	0.3	Fluidresin	6.8E + 09	0.2
Gutta-percha	690	0.45	Composite resin	1.27E + 10	0.35
Periodontal ligament	6.89E + 07	0.45	Adhesive	8.3E + 09	0.35

#### Boundary conditions

To simplify the analysis, the components are assumed to be homogeneous, continuous, and isotropic linear elastic materials. In addition, there is no relative sliding between them.

### Loading mode

Chewing motion in the oral cavity was simulated by static loading. The central fovea of maxillary first premolars was vertically loaded, and the middle triangular ridge of the tongue tip was obliquely loaded. The loading direction was 45° with the dental long axis, and the loading force was 270 N (i.e., maxillary first premolars maximum force).

### Observation Indicators

This experiment focused on comparing the magnitude and distribution of stresses in each com-ponent of endodontically treated maxillary premolar teeth restored with endocrown under different base materials and thicknesses. The main observations were Von-Mises stress (Seqv), also known as equivalent stress, first principal stress (σ1), second principal stress (σ2), and third principal stress (σ3).

## Results

### The maxillary premolar teeth restored with endocrowns under different base thicknesses

## Stress magnitude and distribution of each component in maxillary premolar teeth restored with endocrowns under different base thicknesses (base material is the fluid res-in)

**Fig. 3:**
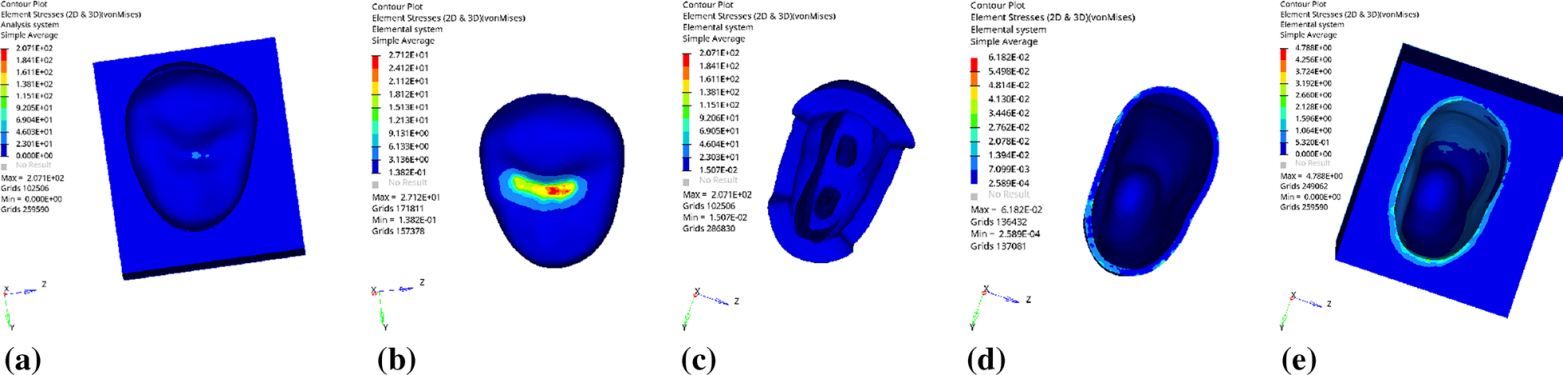
Distribution of equivalent stress for vertical loading without base material: **a** Whole; **b** Endocrown. **c** Residual Tooth. **d** Periodontal Ligament. **e**Alveolar Bone

**Fig. 4:**
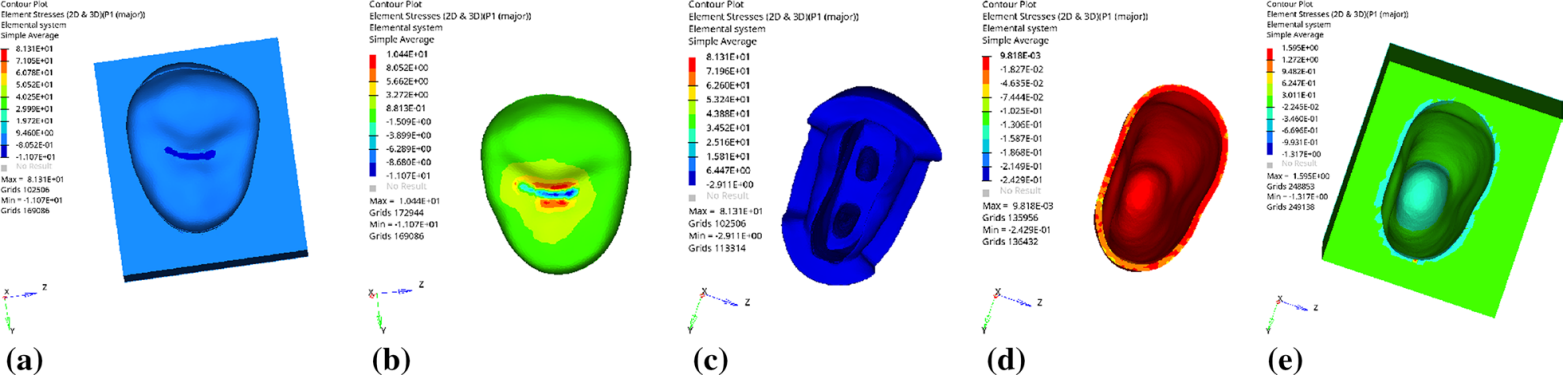
Distribution of the first principal stress without base material under vertical loading: **a** Whole; **b** Endocrown. **c** Residual Tooth. **d** Periodontal Ligament. **e**Alveolar Bone

**Fig. 5:**
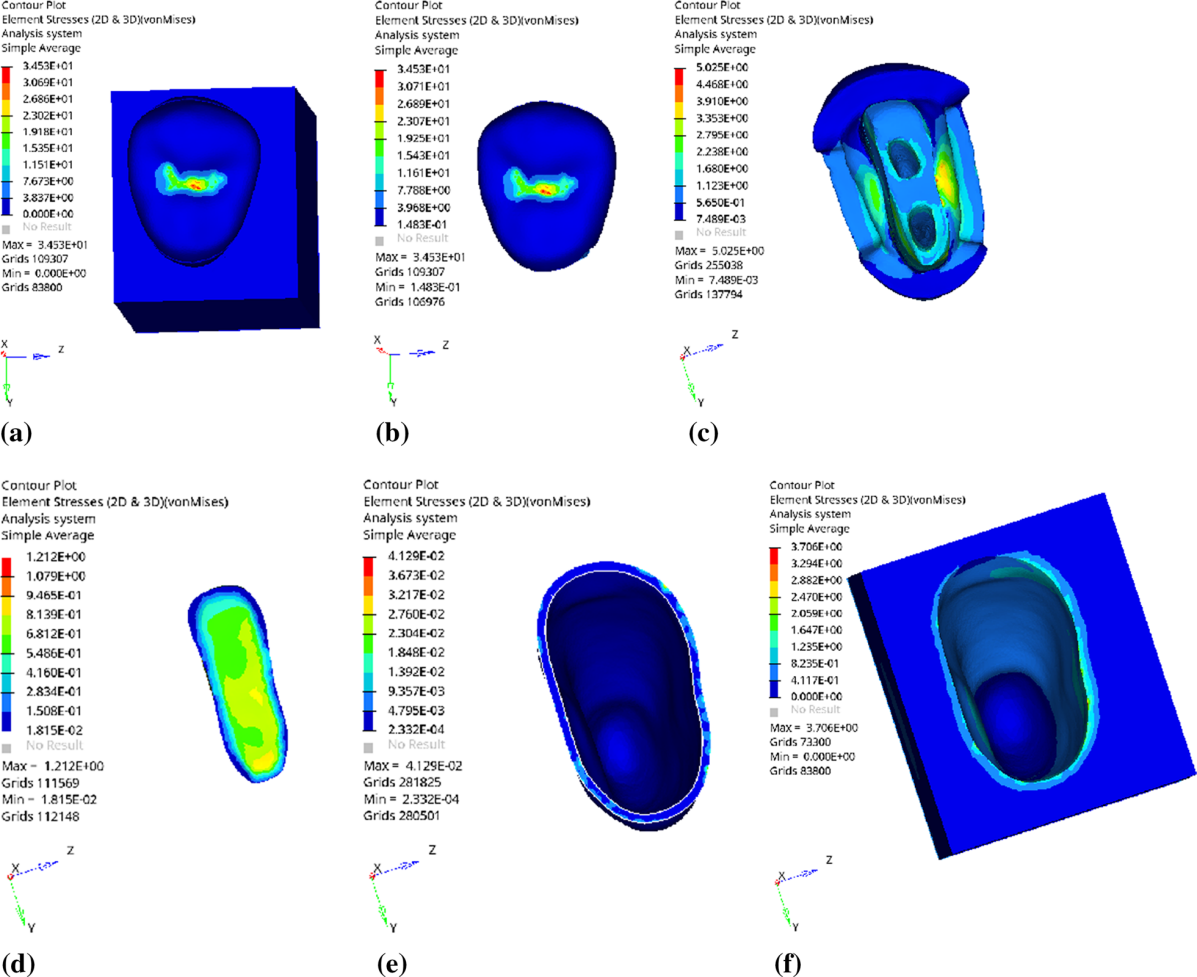
Distribution of equivalent stress of 1 mm fluid resin as base layer under vertical loading: **a** Whole; **b** Endocrown. **c** Residual Tooth. **d** Base layer. **e** Periodontal Ligament. **f**Alveolar Bone

**Fig. 6:**
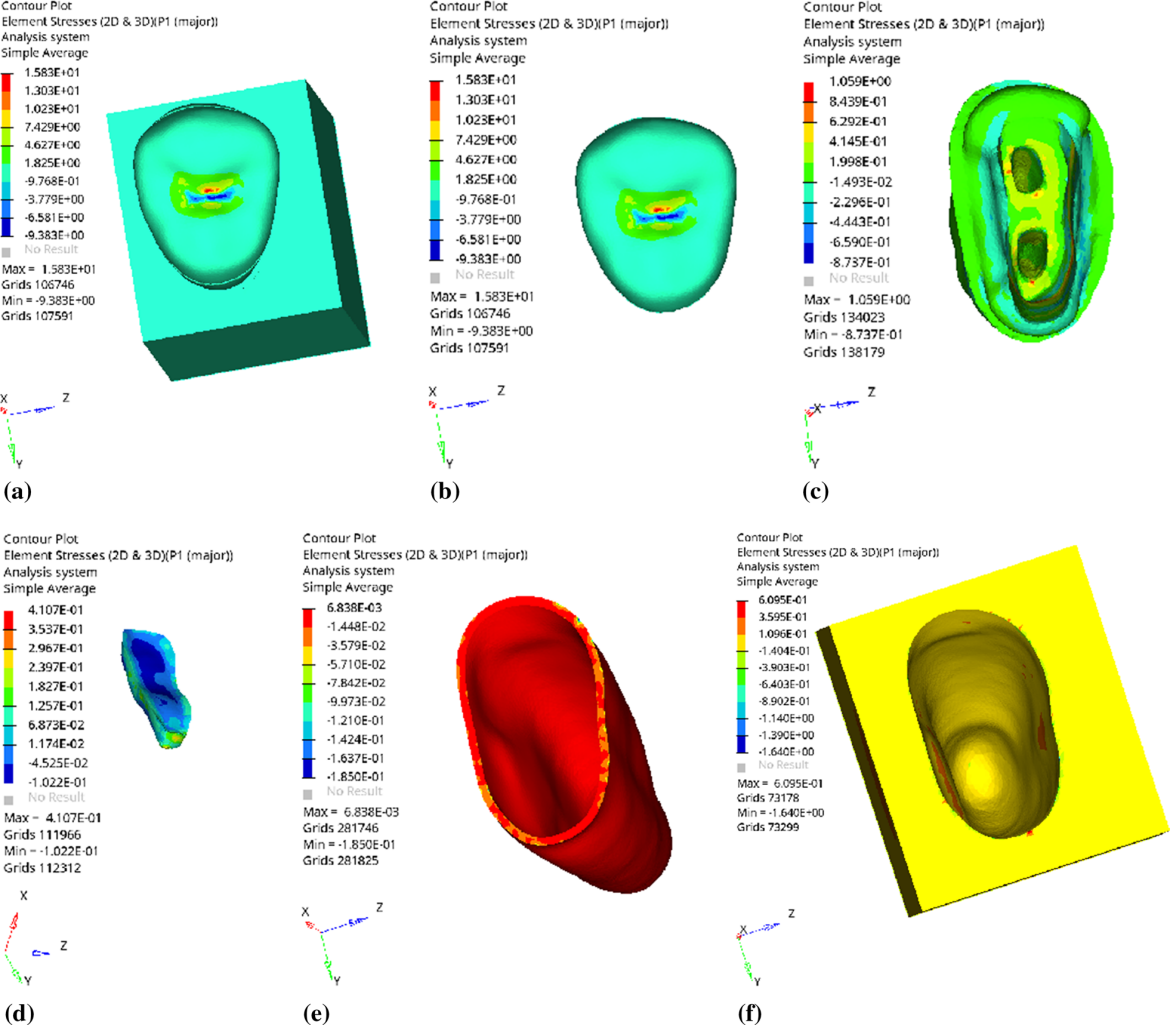
Distribution of first principal stress of 1 mm fluid resin as base layer under vertical loading: **a** Whole; **b** Endocrown. **c** Residual Tooth. **d** Base layer. **e** Periodontal Ligament. **f**Alveolar Bone

**Fig. 7:**
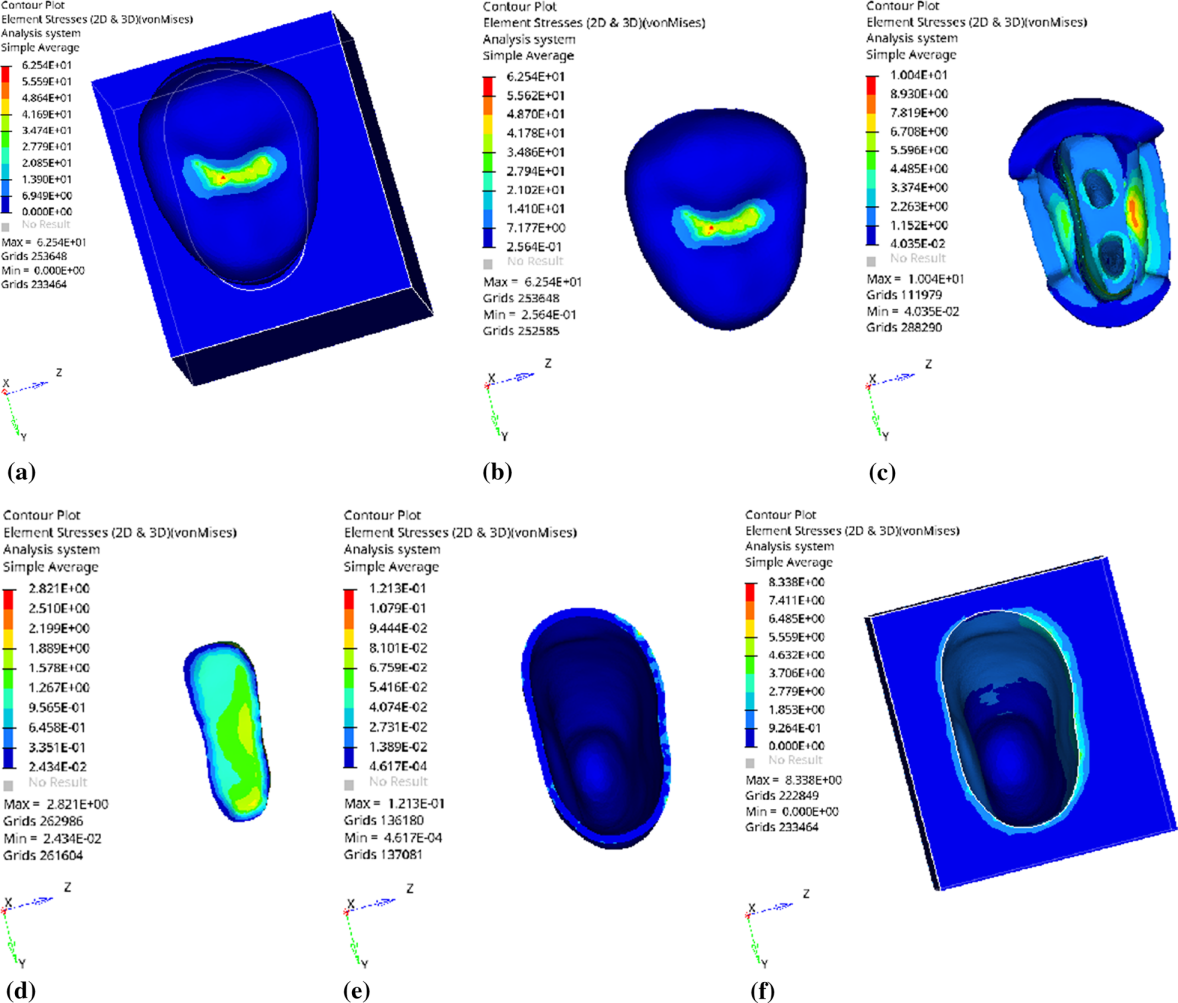
Distribution of equivalent stress of 2 mm fluid resin as base layer under vertical loading: **a** Whole; **b** Endocrown. **c** Residual Tooth. **d** Base layer. **e** Periodontal Ligament. **f** Alveolar Bone

**Fig. 8:**
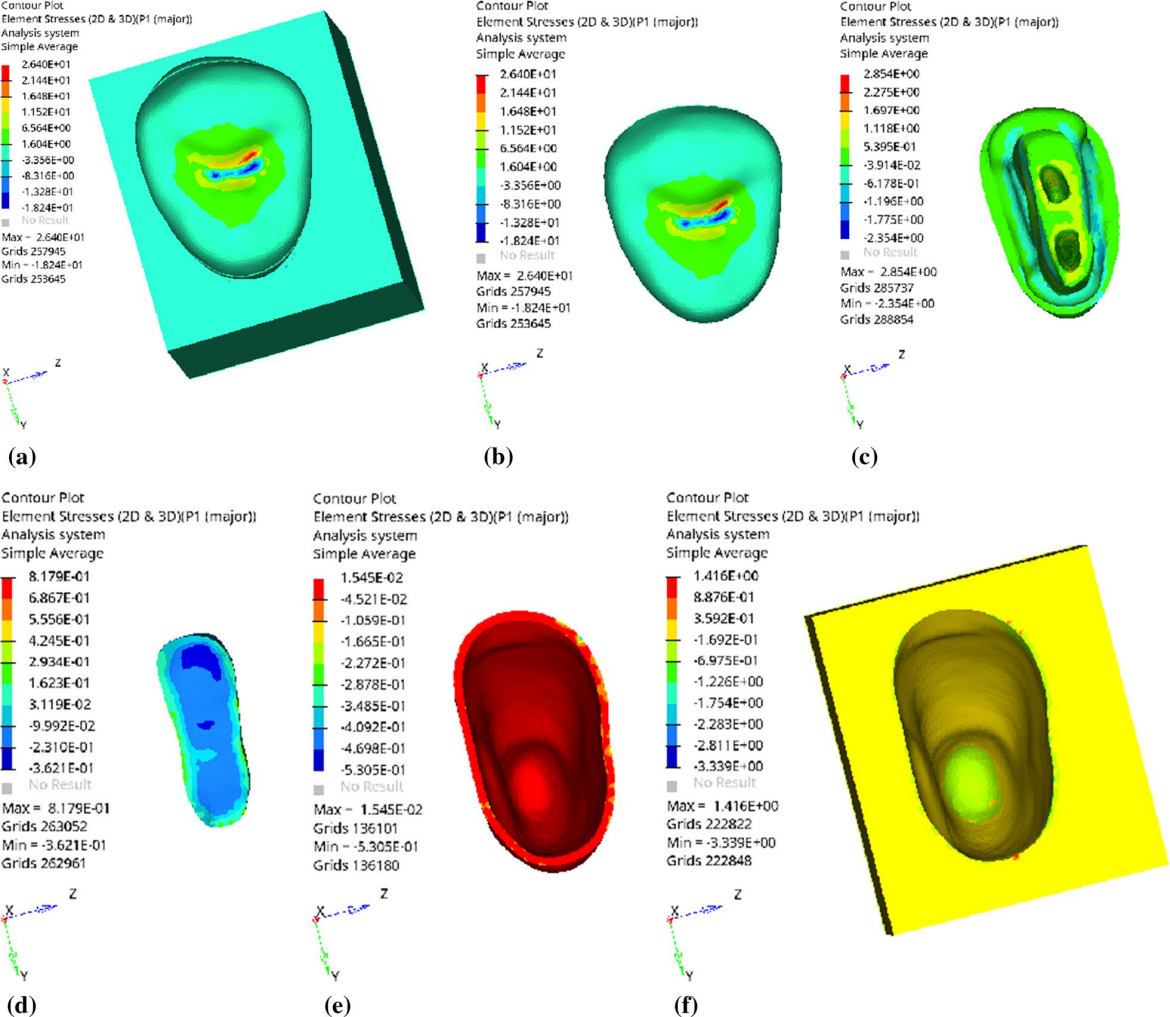
Distribution of the first principal stress of 2 mm fluid resin as base layer under vertical loading: **a** Whole; **b** Endocrown. **c** Residual Tooth. **d** Base layer. **e** Periodontal Ligament. **f** Alveolar Bone

**Fig. 9:**
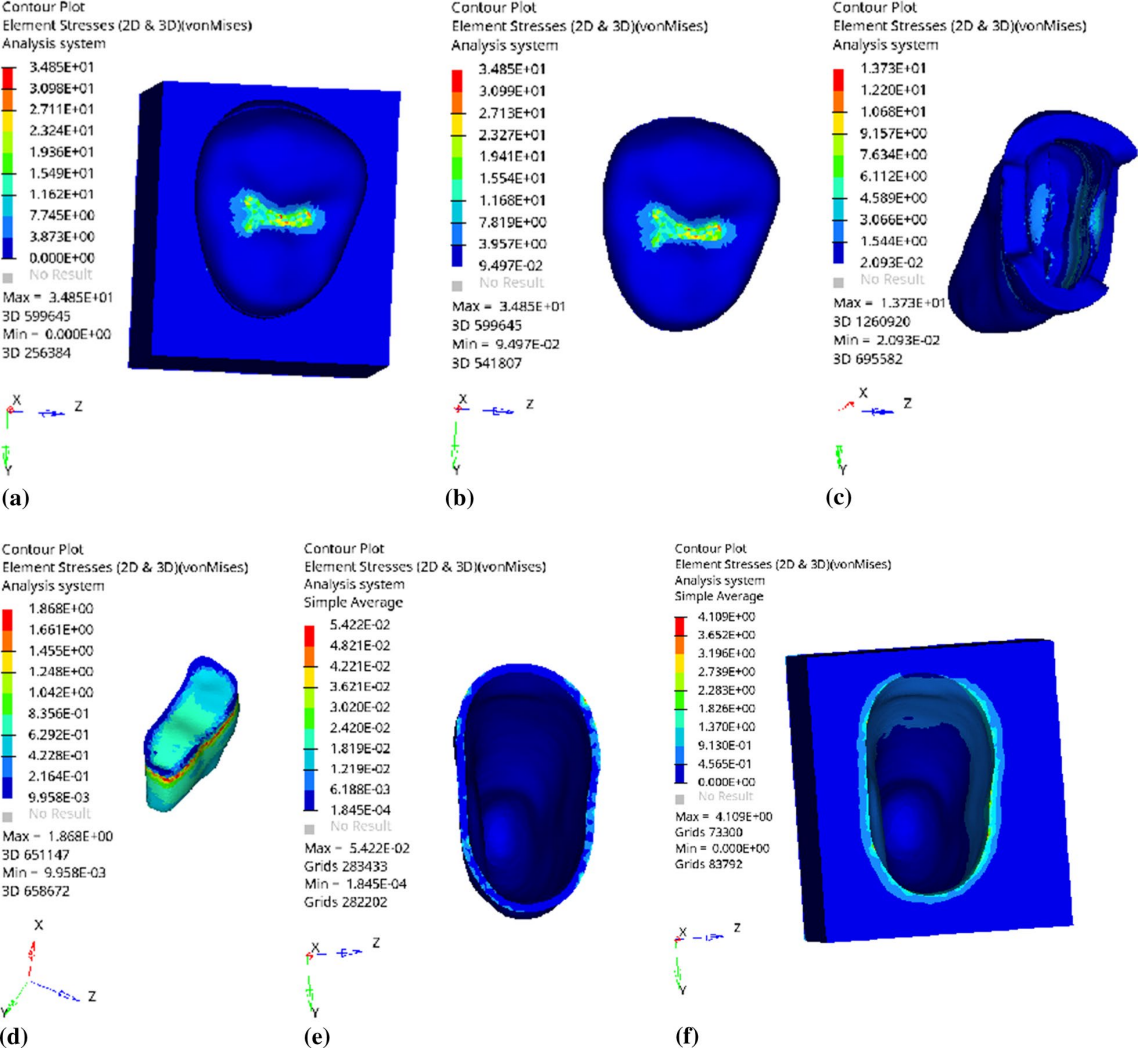
Distribution of equivalent stress of 3 mm fluid resin as base layer under vertical loading: **a** Whole; **b** Endocrown. **c** Residual Tooth. **d** Base layer. **e** Periodontal Ligament. **f** Alveolar Bone

**Fig. 10:**
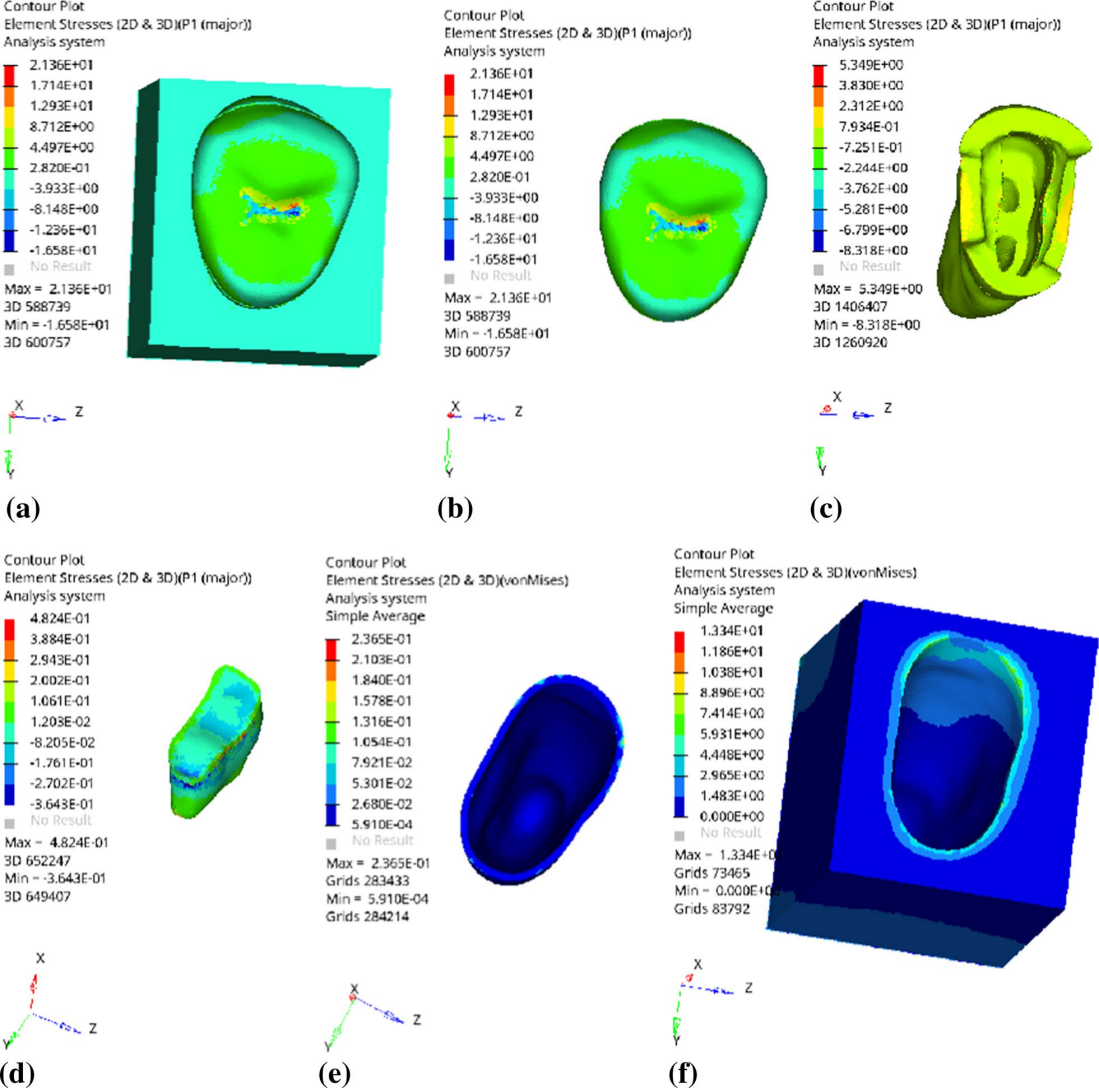
Distribution of the first principal stress of 3 mm fluid resin as base layer under vertical loading: **a** Whole; **b** Endocrown. **c** Residual Tooth. **d** Base layer. **e** Periodontal Ligament. **f**Alveolar Bone

**Fig. 11:**
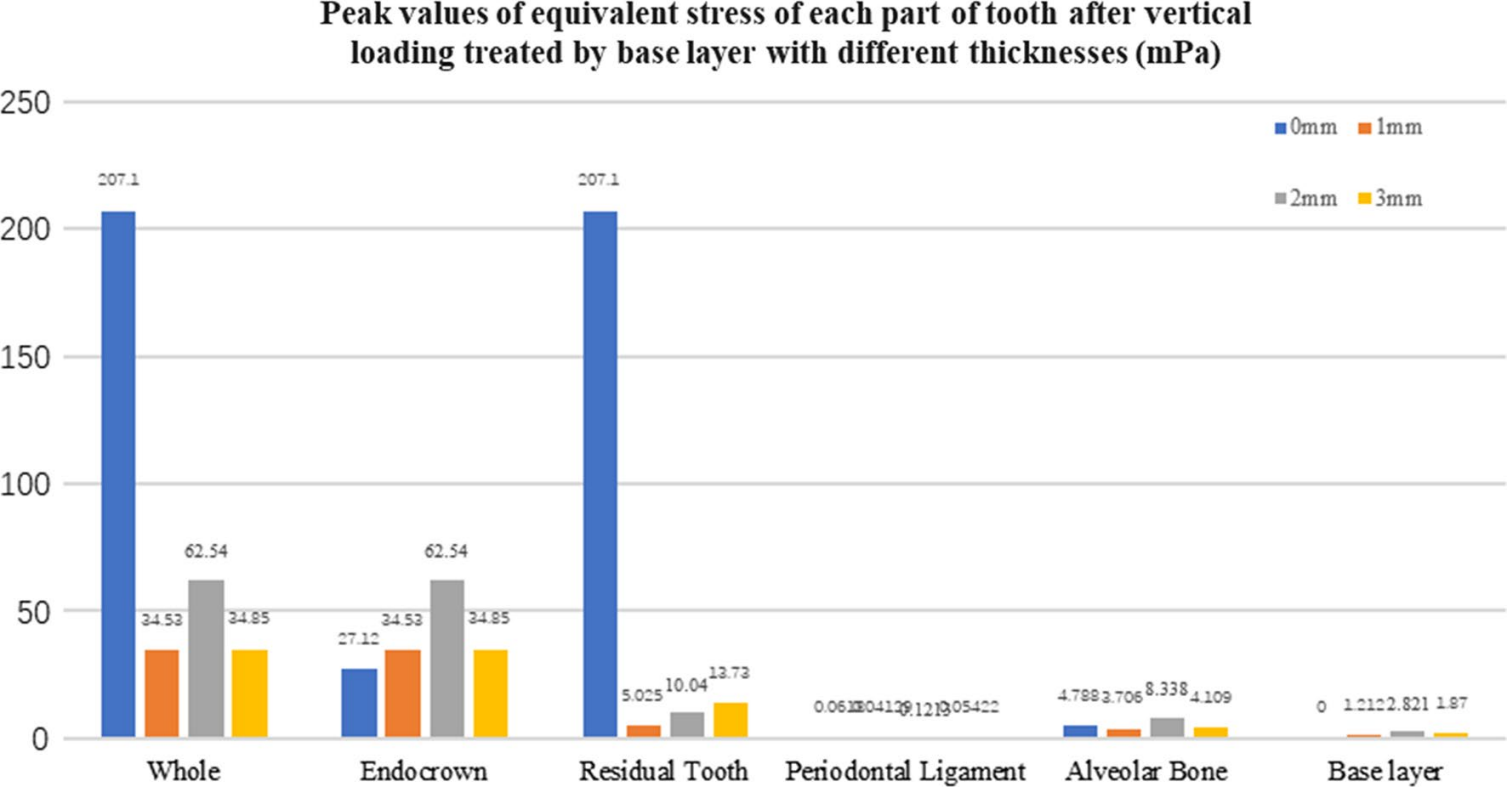
The peak values of equivalent stress (mPa) of each part of the tooth body treated with different thicknesses of base material (fluid resin) under vertical loading

**Fig. 12:**
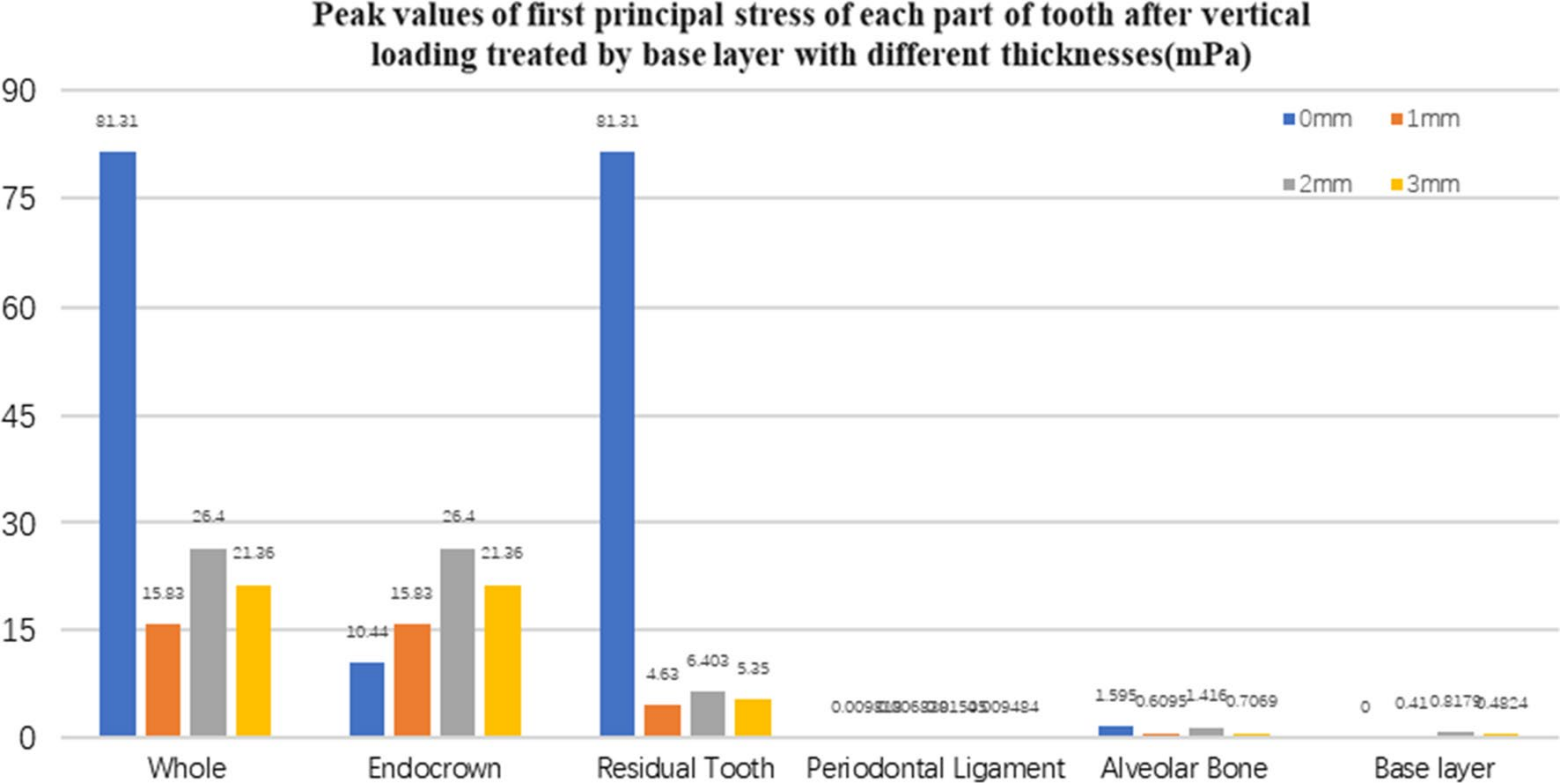
The first principal stress peak (mPa) of each part of the tooth body treated with different thicknesses of base material (fluid resin) under vertical loading

**Fig. 13:**
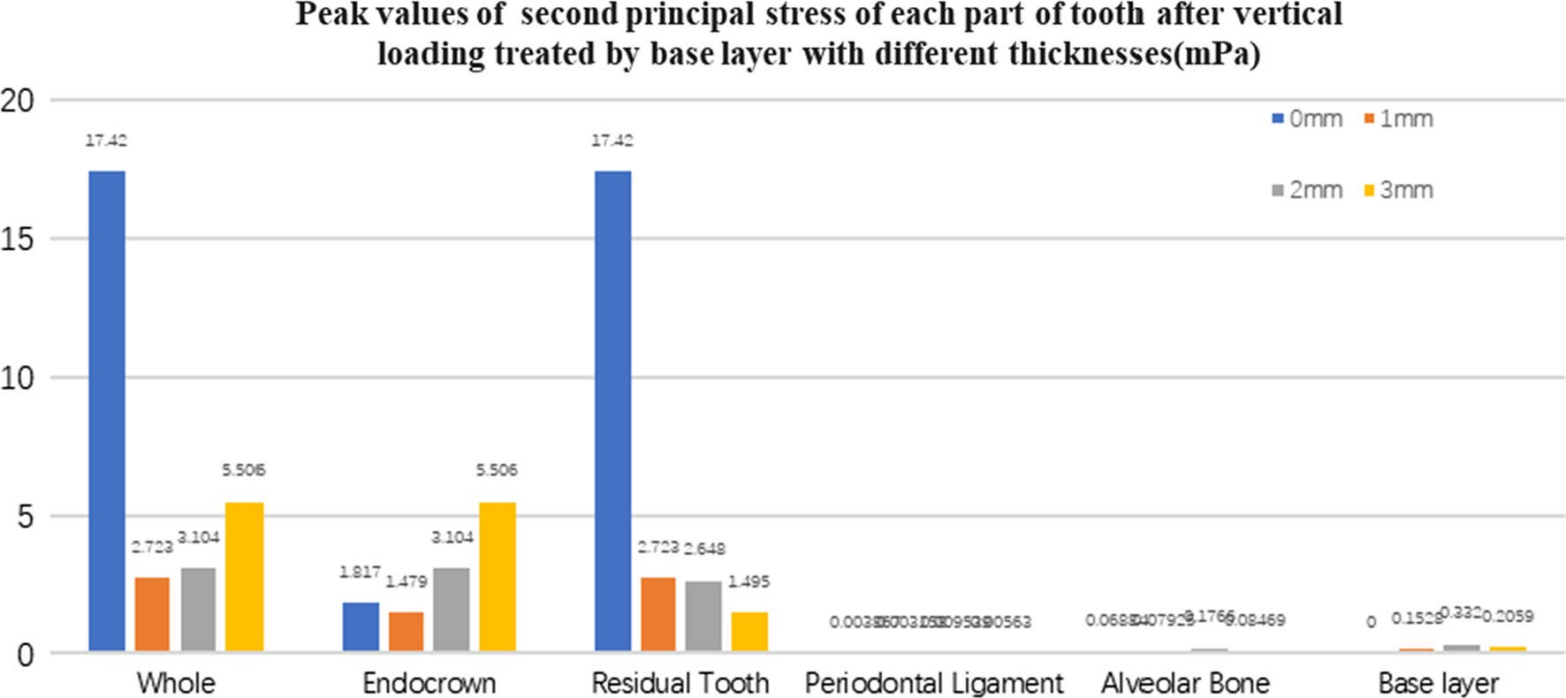
The second principal stress peak (mPa) of each part of the tooth body treated with different thicknesses of base material (fluid resin) under vertical loading

**Fig. 14:**
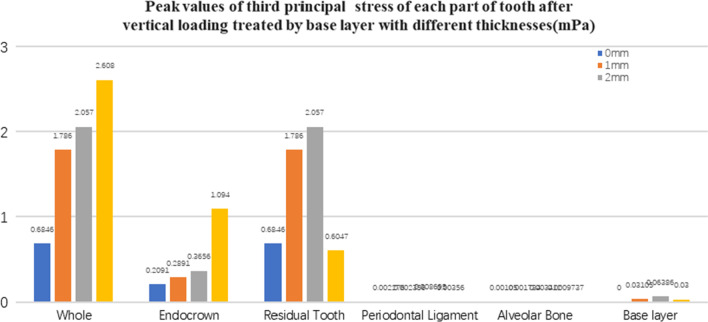
The third principal stress peak (mPa) of each part of the tooth body treated with different thicknesses of base material (fluid resin) under vertical loading

**Fig. 15:**
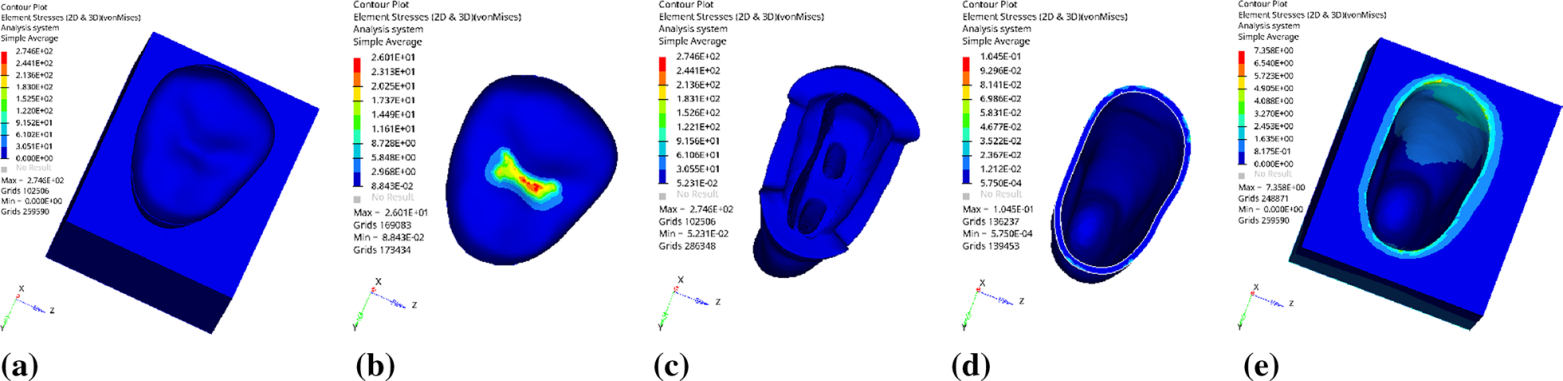
Distribution of equivalent stress for Oblique Loading without base material: **a** Whole; **b** Endocrown. **c** Residual Tooth. **d** Periodontal Ligament. **e** Alveolar Bone

**Fig. 16:**
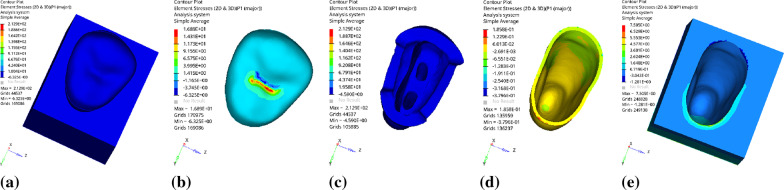
Distribution of the first principal stress for oblique loading without base material: **a** Whole; **b** Endocrown. **c** Residual Tooth. **d** Periodontal Ligament. **e** Alveolar Bone

**Fig. 17:**
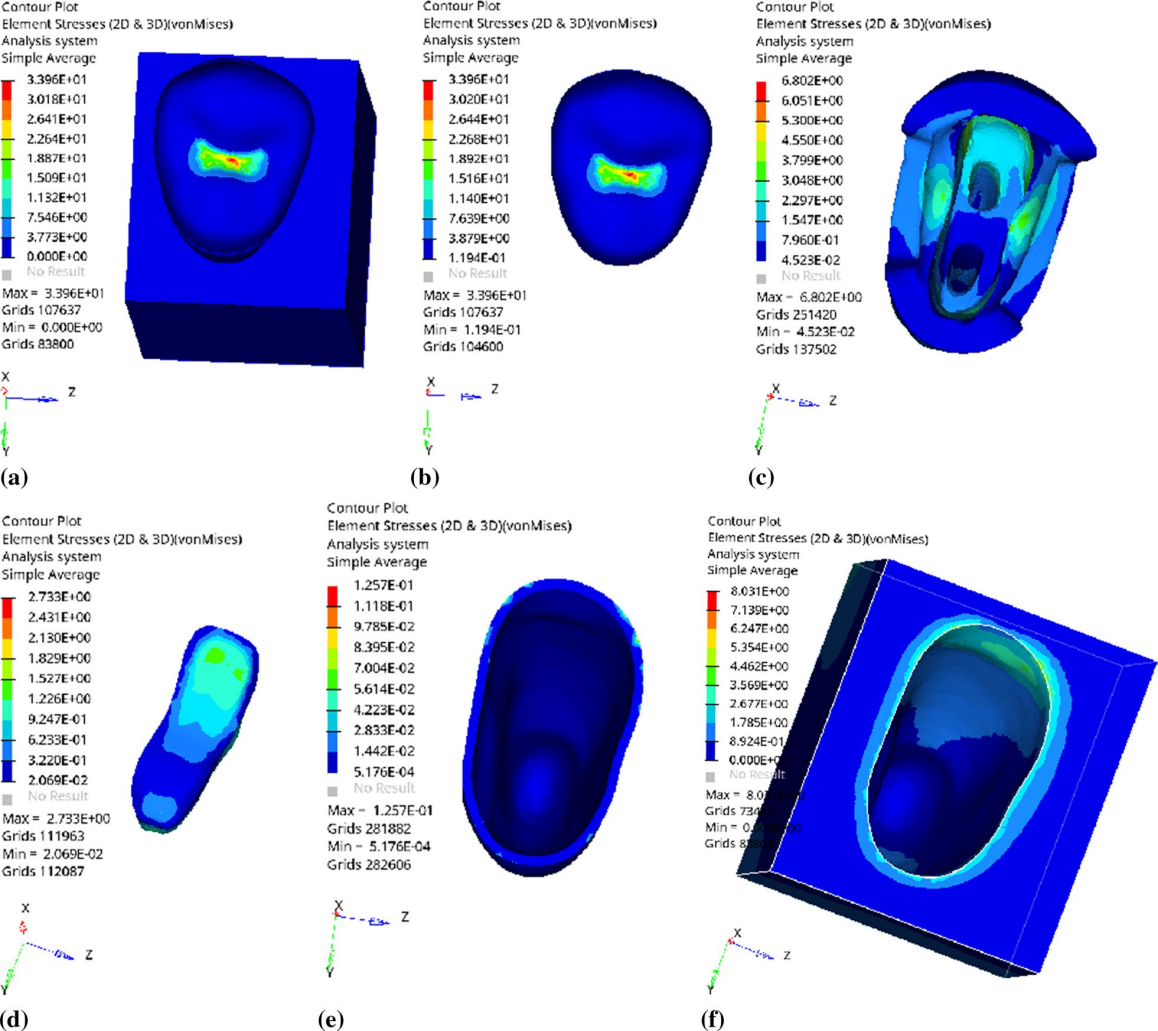
Distribution of equivalent stress of 1 mm fluid resin as base layer under oblique loading: **a** Whole; **b** Endocrown. **c** Residual Tooth. **d** Base layer. **e** Periodontal Ligament. **f** Alveolar Bone

**Fig. 18:**
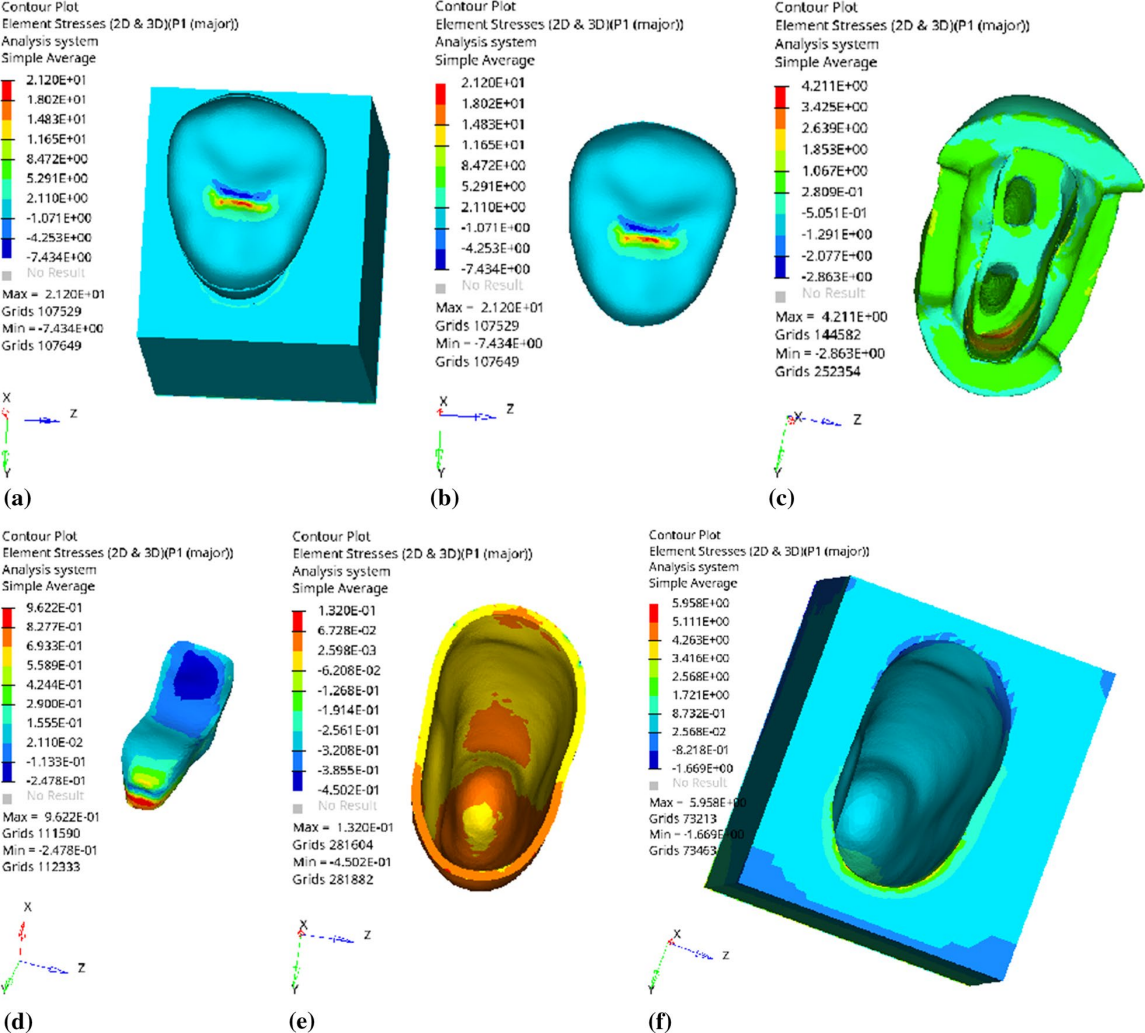
Distribution of first principal stress of 1 mm fluid resin as base layer under oblique loading: **a** Whole; **b** Endocrown. **c** Residual Tooth. **d** Base layer. **e** Periodontal Ligament. **f** Alveolar Bone

**Fig. 19:**
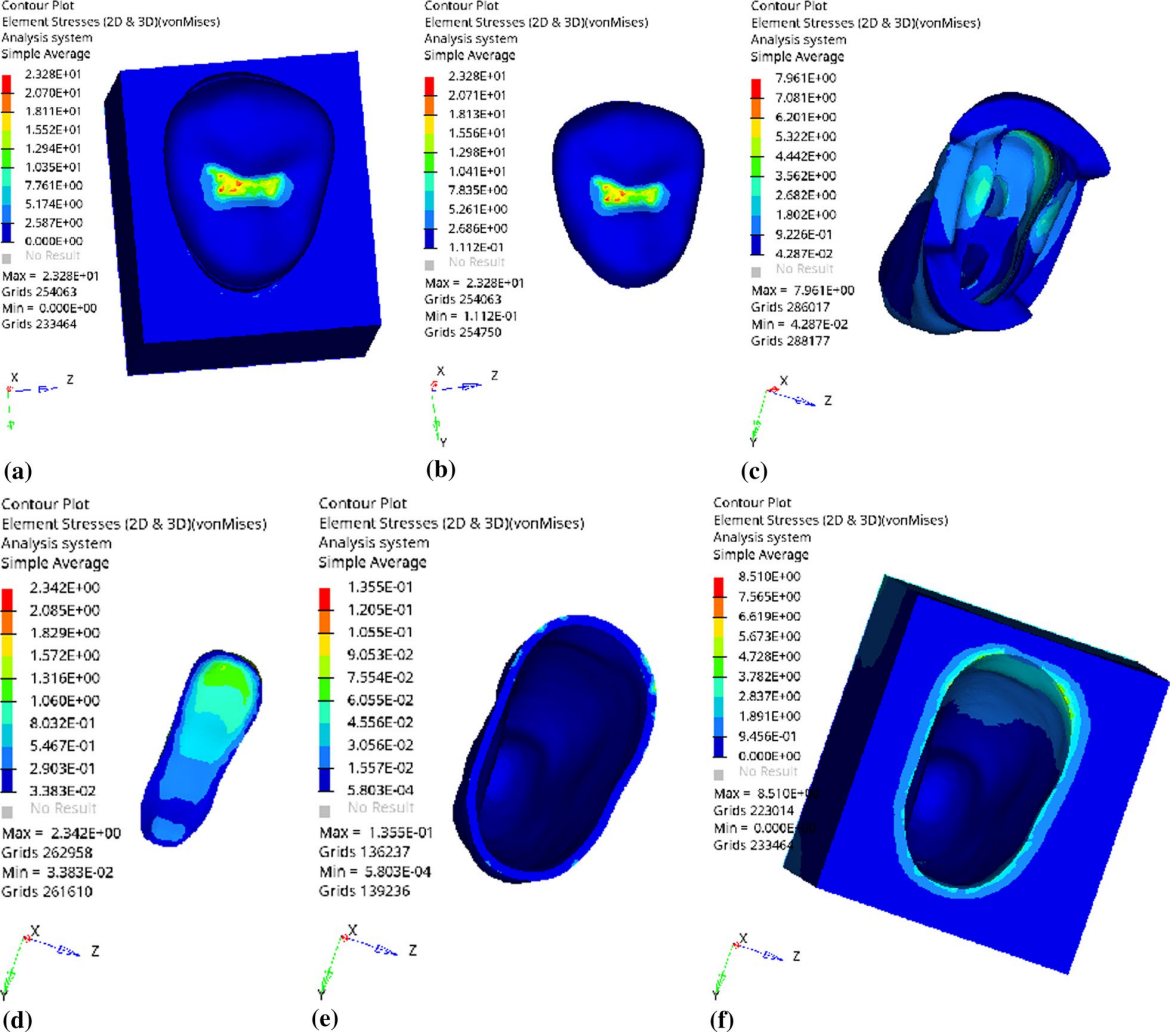
Distribution of equivalent stress of 2 mm fluid resin as base layer under oblique loading: **a** Whole; **b** Endocrown. **c** Residual Tooth. **d** Base layer. **e** Periodontal Ligament. **f** Alveolar Bone

**Fig. 20:**
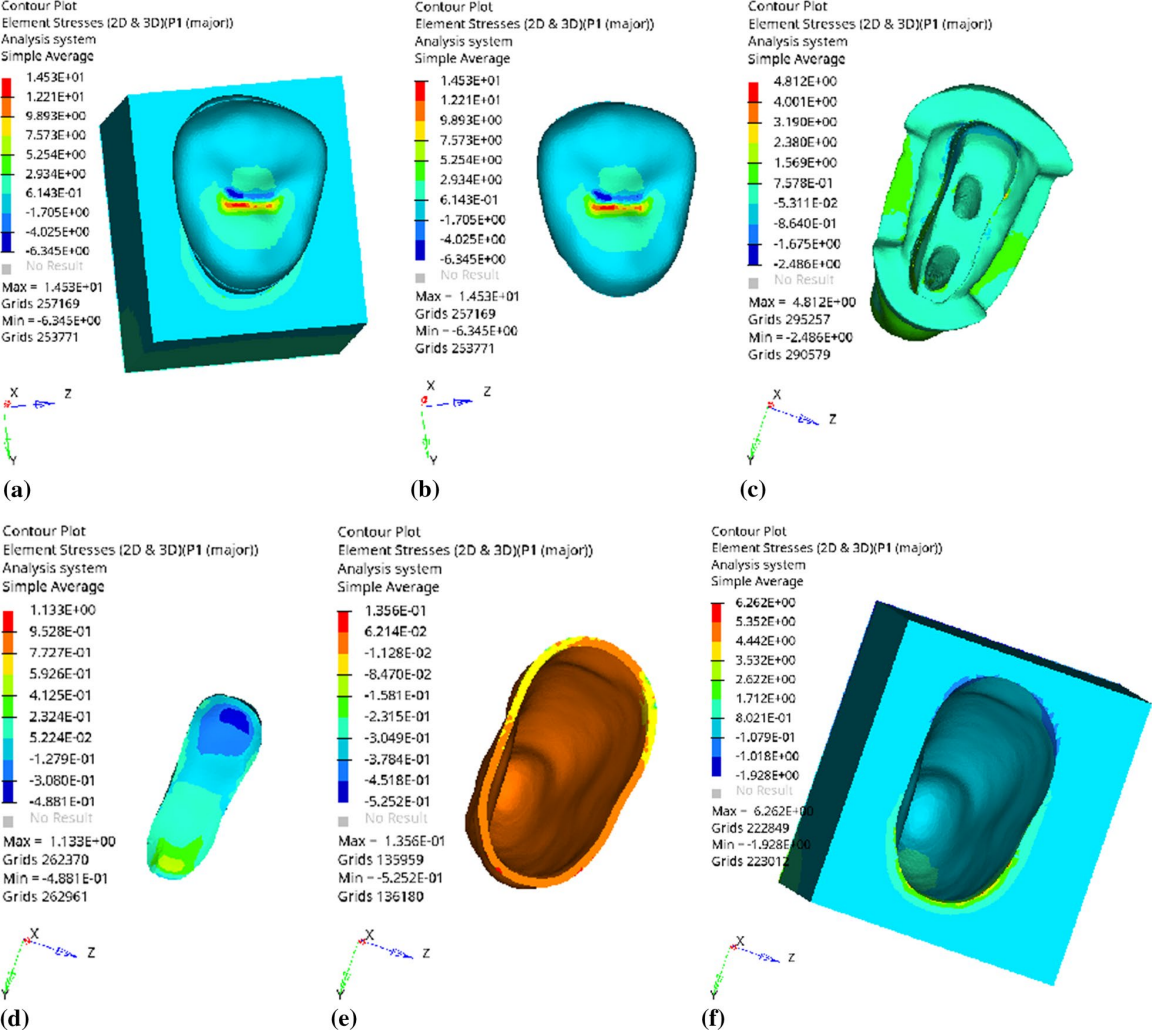
Distribution of the first principal stress of 2 mm fluid resin as base layer under oblique loading: **a** Whole; **b** Endocrown. **c** Residual Tooth. **d** Base layer. **e** Periodontal Ligament. **f** Alveolar Bone

**Fig. 21:**
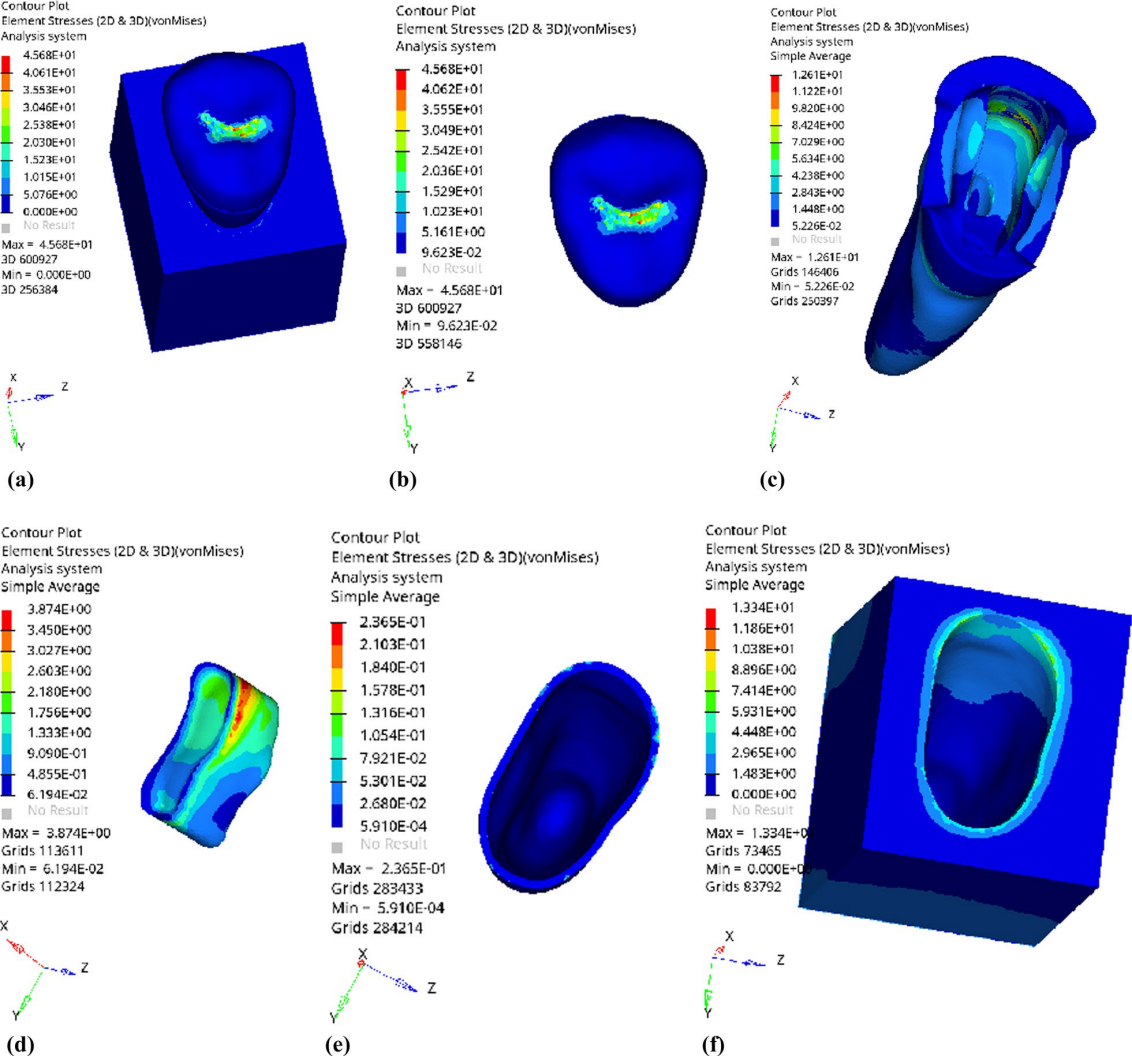
Distribution of equivalent stress of 3 mm fluid resin as base layer under oblique loading: **a** Whole; **b** Endocrown. **c** Residual Tooth. **d** Base layer. **e** Periodontal Ligament. **f** Alveolar Bone

**Fig. 22:**
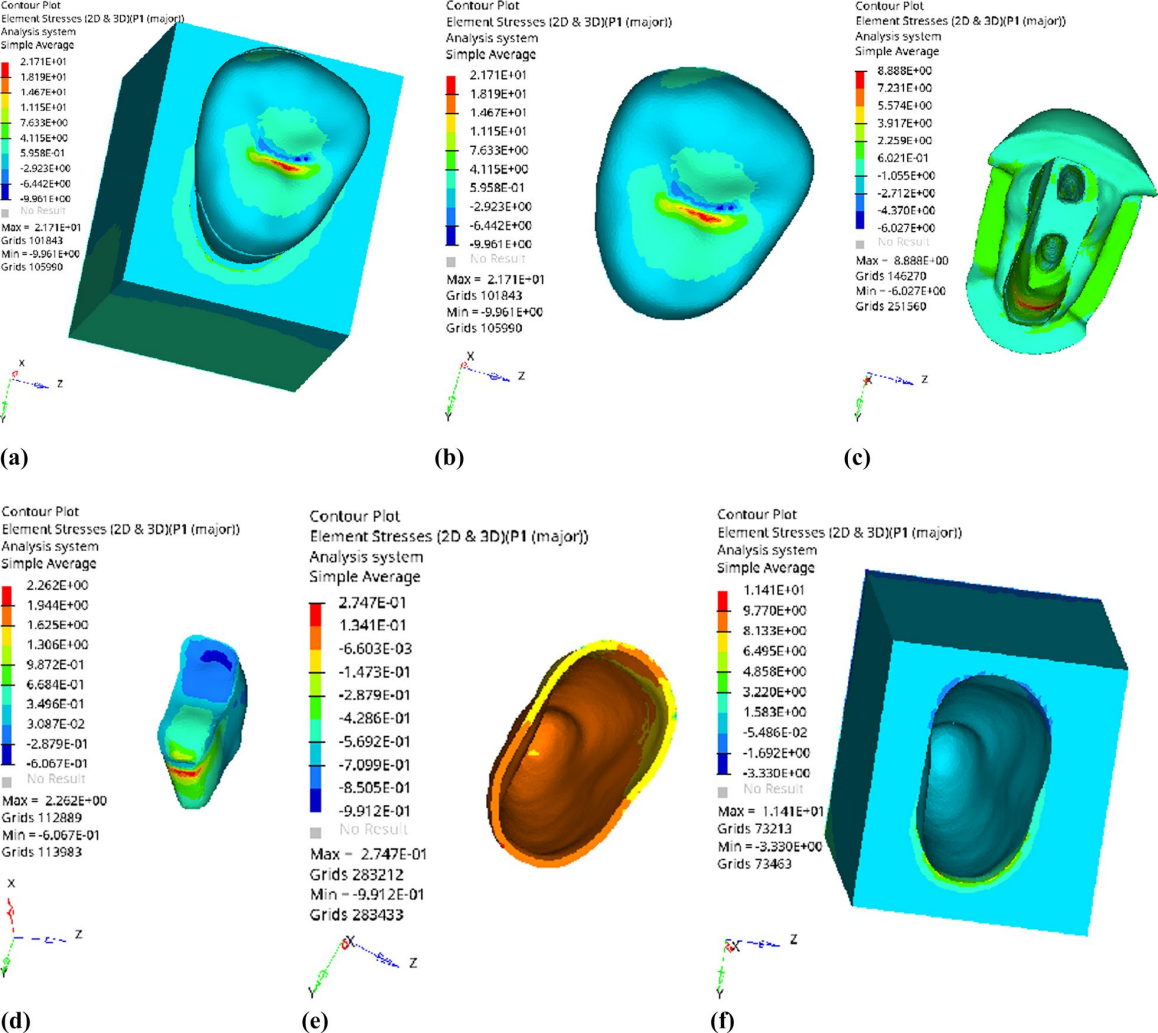
Distribution of the first principal stress of 3 mm fluid resin as base layer under oblique loading: **a** Whole; **b** Endocrown. **c** Residual Tooth. **d** Base layer. **e** Periodontal Ligament. **f** Alveolar Bone

**Fig. 23:**
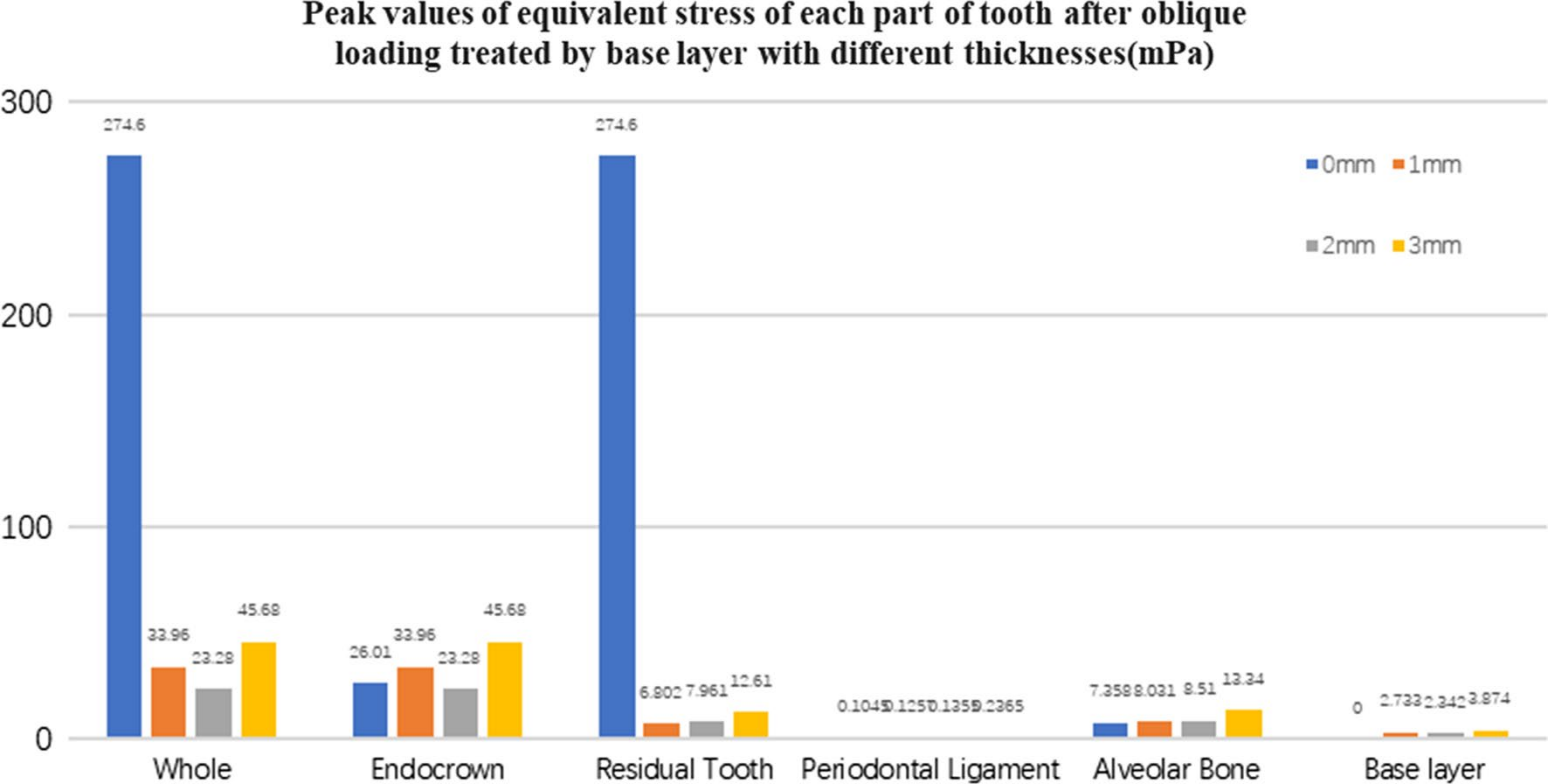
The equivalent stress peak value (mPa) of each part of tooth body treated with different thickness of base materials (fluid resin) under oblique loading

**Fig. 24:**
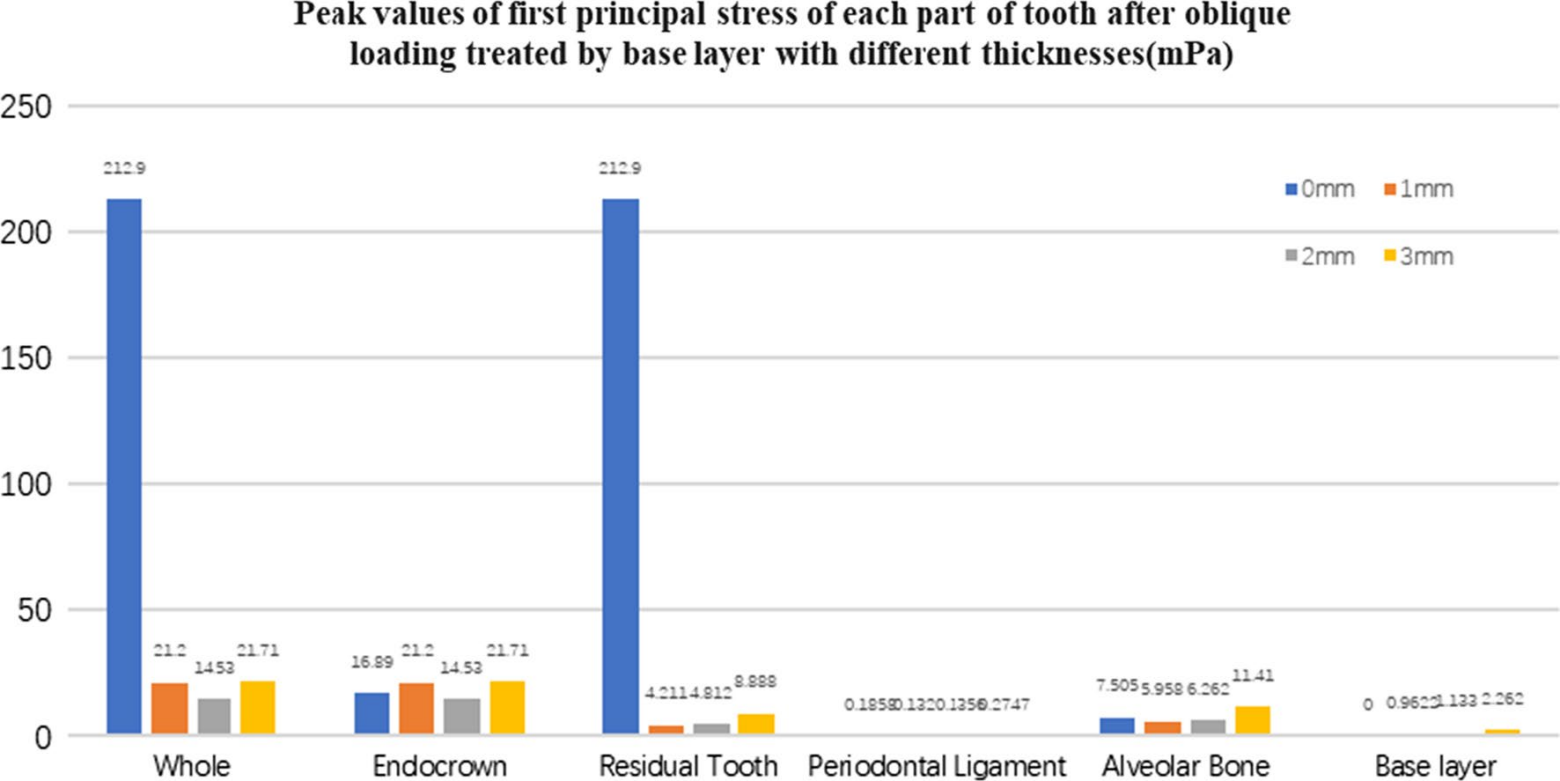
The first stress peak value (mPa) of each part of tooth body treated with different thickness of base materials (fluid resin) under oblique loading

**Fig. 25:**
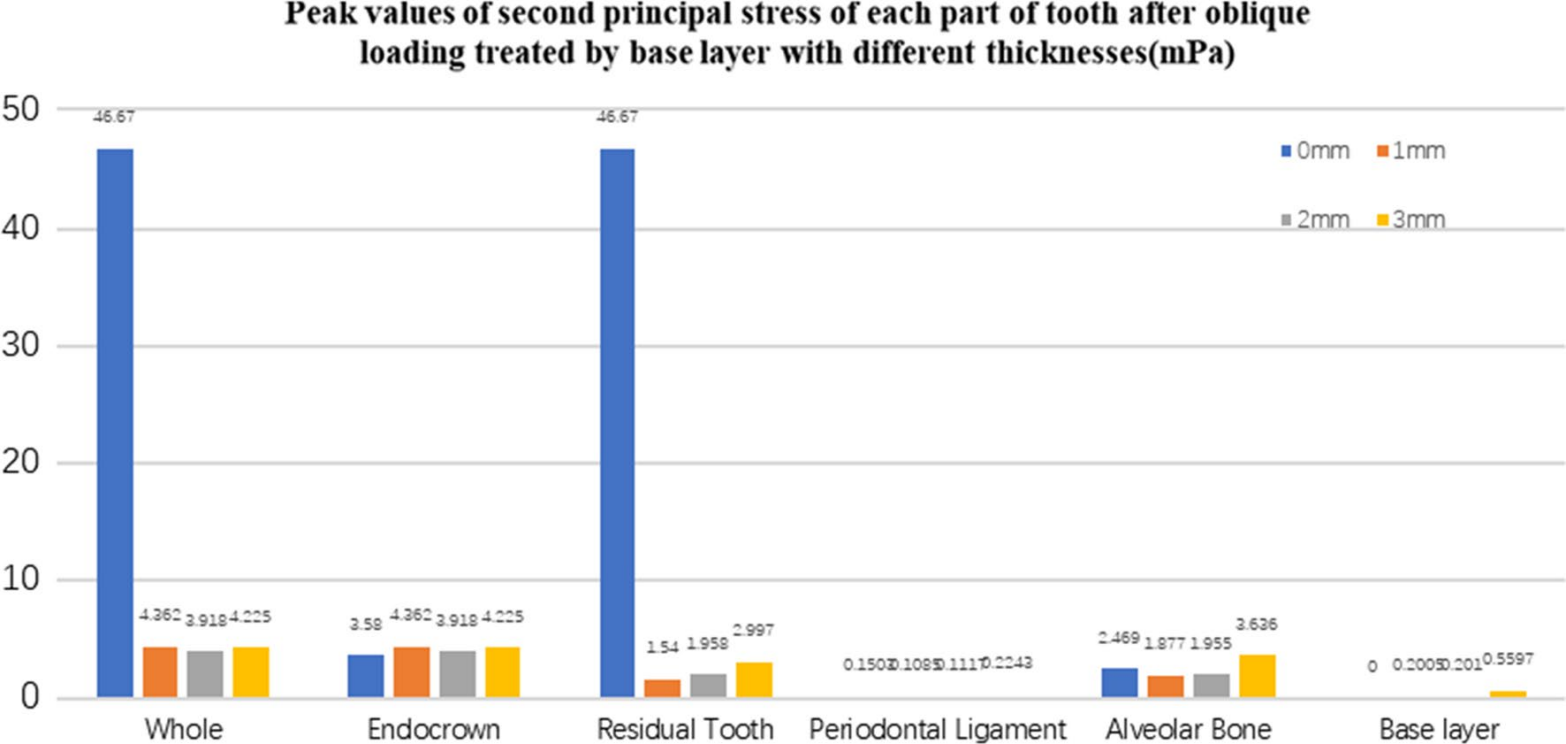
The second stress peak value (mPa) of each part of tooth body treated with different thickness of base materials (fluid resin) under oblique loading

**Fig. 26:**
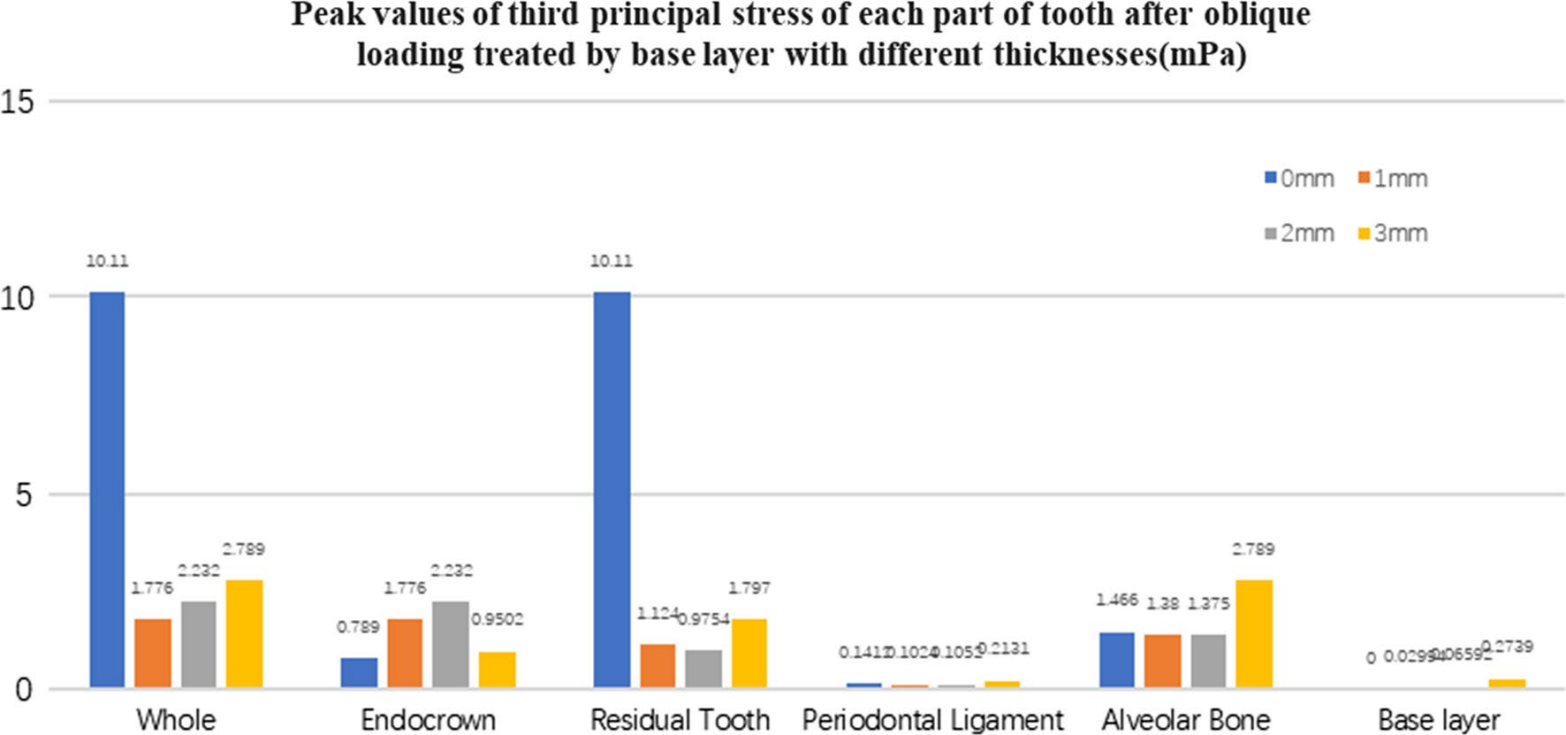
The third stress peak value (mPa) of each part of tooth body treated with different thickness of base materials (fluid resin) under oblique loading

### Analysis of stress distribution of each component with different base thicknesses (the base material is fluid resin)

Since the strain and stress distribution of the restorations and dentition restored with endocrowns under different base thicknesses were similar, the equivalent stress and first principal stress distribution when the base material was fluid resin were selected as representative. Among them, Figs. 3, 4, 5, 6, 7, 8, 9 and 10 show the loading results of the restorations and various parts of the dentition in the axial direction of 270 N. Figures 11, 12, 13, 14 show the trends in stress values of different stress (mPa) of each part of the tooth body treated with different thicknesses of base material under vertical loading; Figs. 15, 16, 17, 18, 19, 20, 21 and 22 show the loading results of 270 N after an oblique 45°.

1. The distribution of the equivalent stress (Von-Mises), the first principal stress (σ_1_), the second principal stress (σ_2_), and the third principal stress (σ_3_) during axial loading is as follows:

*Overall stress* The peak equivalent stress without base layer is located in the lingual distal center of the dentoenamel junction. The peak equivalent stress is located in the remote central fovea distal of the endocrown for 1 mm, 2 mm and 3 mm base thickness. The peak of σ_1_ is located at the lingual distal center of the dentoenamel junction without the base layer. The peak of σ_1_ is located in the buccal cusp of the central fovea of the endocrown when the base thickness is 1 mm, 2 mm, and 3 mm. σ_2_ and σ_3_ principal stresses are more uniformly distributed;

*Endocrown* The peak equivalent stress is larger at the loading point (i.e., the central fovea), and the peak is slightly towards the distal of the central fovea; σ_1_, σ_2_, and σ_3_ peaks are mostly located near the central fovea.

Base layer The peak equivalent stress is located on the contact surface of the lingual side when the thickness of the base layer is 1 mm; the peak equivalent stress is located in the proximal 1/3 area of the lingual side when it is 2 or 3 mm; σ1, σ2, and σ3 are small, and the lingual side is relatively large.

Residual tooth The peak of the equivalent stress, σ1, and σ2 are located in the remote lingual side of the dentoenamel junction at the base thicknesses of 0, 2 and 3 mm, and in the proximal middle 1/2 of the endocrown contact surface at the base thickness of 1 mm; σ3 is smaller and slightly larger on the distal middle side.

*Periodontal ligament and alveolar bone* The equivalent stress and the first, second and third principal stress distribution are relatively uniform; the stress value is small and slightly larger than the tongue.

2. Under 45° oblique loading, the distribution of equivalent stress (Von-Mises), first principal stress (σ_1_), second principal stress (σ_2_) and third principal stress (σ_3_) is as follows:

*Overall stress* The peak equivalent stress is located in the lingual distal center of the dentoenamel junction without a base layer and the central fossa with a base thickness of 1 and 3 mm. The peak equivalent stress is located in the middle of the central fovea at 2 mm base thickness. σ1, σ2, and σ3 peaks are similar to the peak equivalent stress distribution.

*Endocrown* The peak equivalent stress is located in the central fovea when the base thickness is 0 mm and 1 mm. When the base thickness was 2 mm and 3 mm, the peak value was located in the proximal and remote fovea, respectively. The distribution of peak σ_1_ is similar to that of equivalent stress. σ_2_ and σ_3_ are small, and the peak values are primarily distributed in fovea centralis.

*Base layer* The peak equivalent stress is located in the middle 1/2 of the buccal margin at a base thickness of 1 mm, in the proximal middle 1/3 of the buccal region at 2 mm, and the proximal middle of the lingual margin at 3 mm; σ_1_, σ_2_, and σ_3_ are small, and the lingual side is relatively large.

*Residual tooth* The peak equivalent stress is located in the lingual distal middle of the dentoenamel junction without a base layer. The peak stress is located in the mid-buccal 1/3 of the dentoenamel junction when the base thickness is 1, 2, and 3 mm. σ_1_, σ_2_, and σ_3_ peaks are mostly distributed in the remote lingual marginal crest of the dentoenamel junction.

*Periodontal ligament and alveolar bone* the distribution of equivalent stress and the first, second and third principal stresses is relatively uniform, the stress value is small, the crown is larger than the apical.

#### Trends in stress values of residual teeth, restorations, and base layers at different thicknesses (Figs. 11, 12, 13,14, 23, 24, 25 and 26)

In the four model groups, the equivalent stress, first principal stress (σ_1_), second principal stress (σ_2_) and third principal stress (σ_3_) of the restoration, residual teeth, base layer, periodontal ligament, and alveolar bone had little difference when the base thickness was 1 mm, 2 mm and 3 mm. However, the stresses on the whole and the residual teeth of the model without the base layer were significantly higher than those of the three groups with the base layer. The stresses on the restoration, periodontal ligament, and alveolar bone had little difference with the other three groups.

It is observed that under vertical loading, the equivalent stress and σ_1_ of the model as a whole are: the base layer is 1 mm < 3 mm < 2 mm < 0 mm, σ_2_ and σ_3_ are small, and the difference is negligible. Under oblique loading, the equivalent stress of the model as a whole and σ_1_: the base layer 2 mm < 1 mm < 3 mm < 0 mm, σ_2_ and σ_3_ are small, and the difference is small.

### The maxillary premolar restored with endocrowns under different base materials

#### Comparison of stress value trends in residual teeth, restorations, and base layers with different base materials (Figs. 27, 28, 29, 30, 31, 32, 33 and 34)

**Fig. 27:**
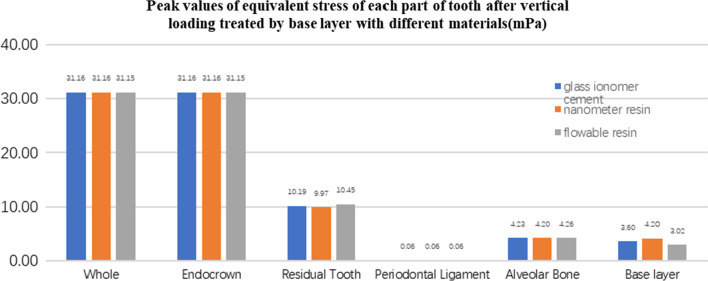
The equivalent stress peak (mPa) of each part of the tooth body treated with different base materials under vertical loading

**Fig. 28:**
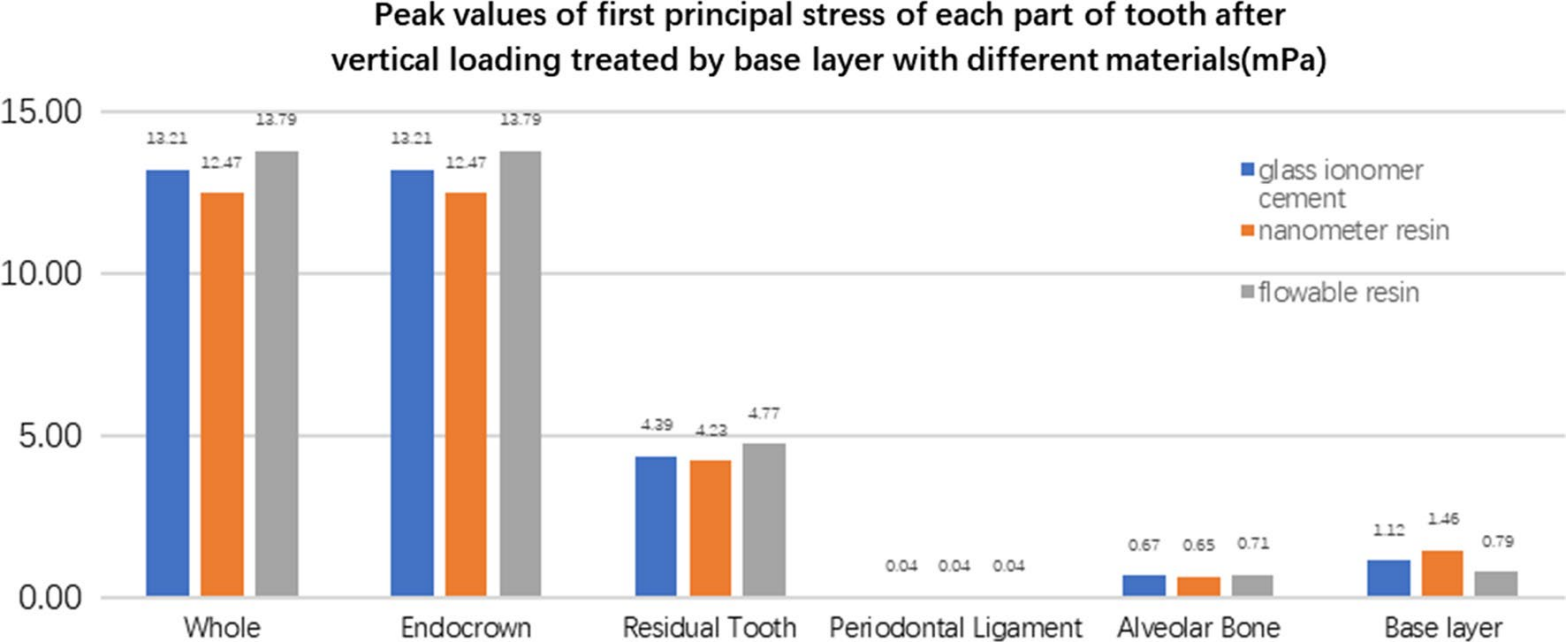
The first stress peak (mPa) of each part of the tooth body treated with different base materials under vertical loading

**Fig. 29:**
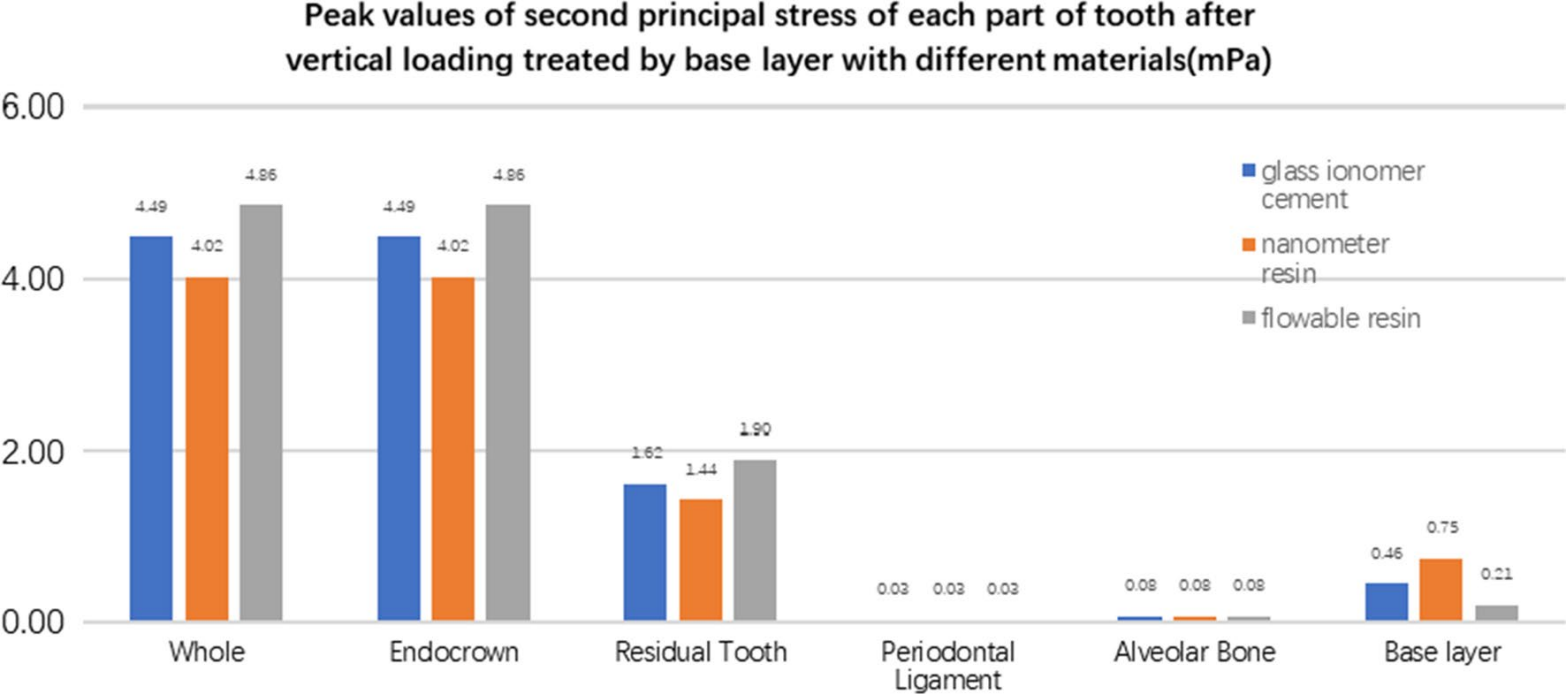
The second stress peak (mPa) of each part of the tooth body treated with different base materials under vertical loading

**Fig. 30:**
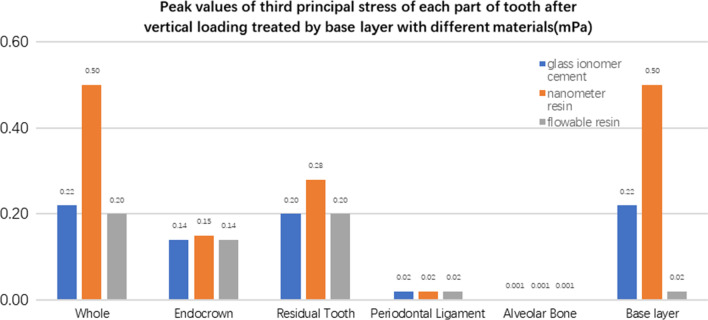
The third stress peak (mPa) of each part of the tooth body treated with different base materials under vertical loading

**Fig. 31:**
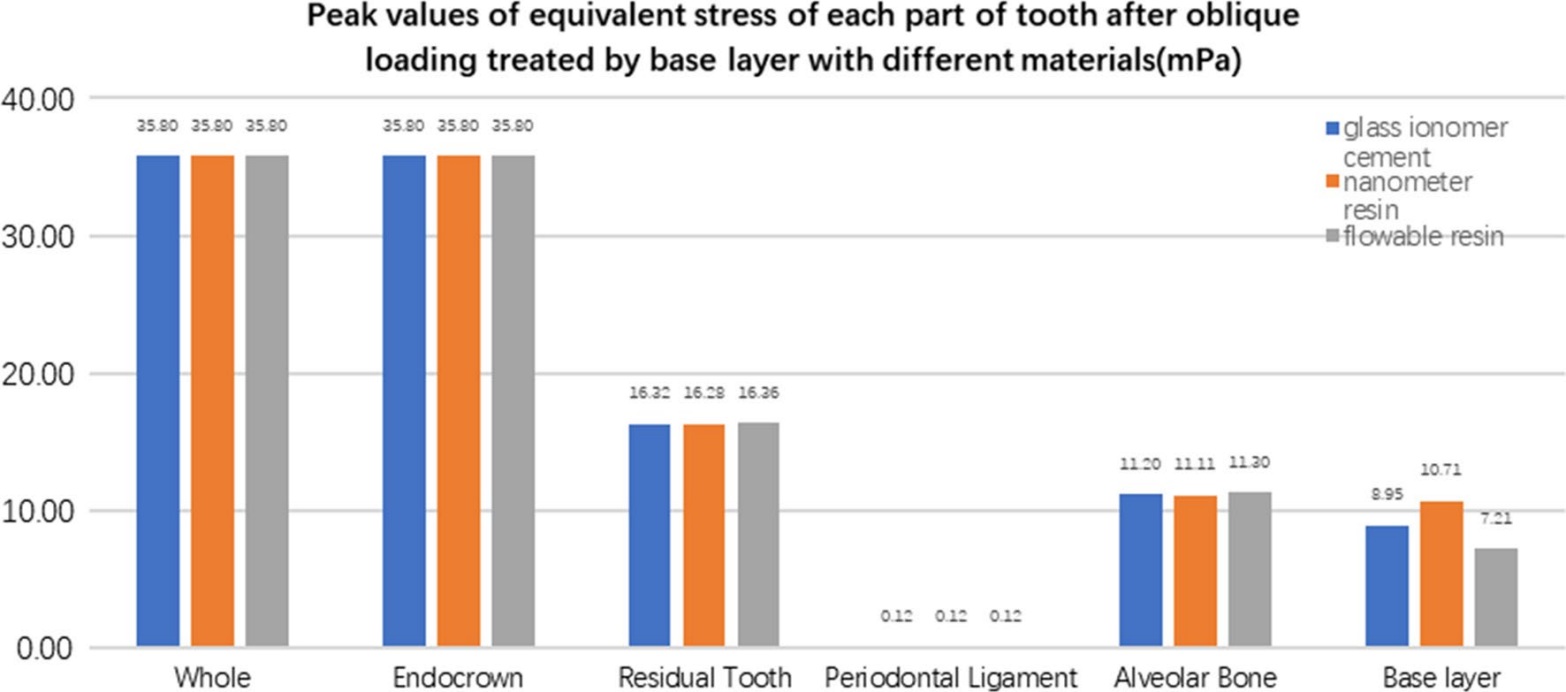
The equivalent stress peak (mPa) of each part of the tooth body treated with different bedding materials under oblique loading

**Fig. 32:**
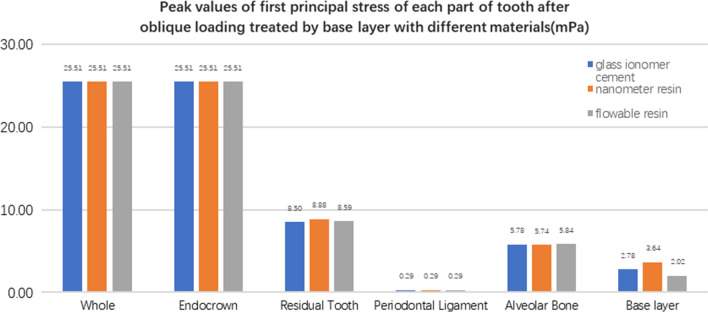
The first stress peak (mPa) of each part of the tooth body treated with different bedding materials under oblique loading

**Fig. 33:**
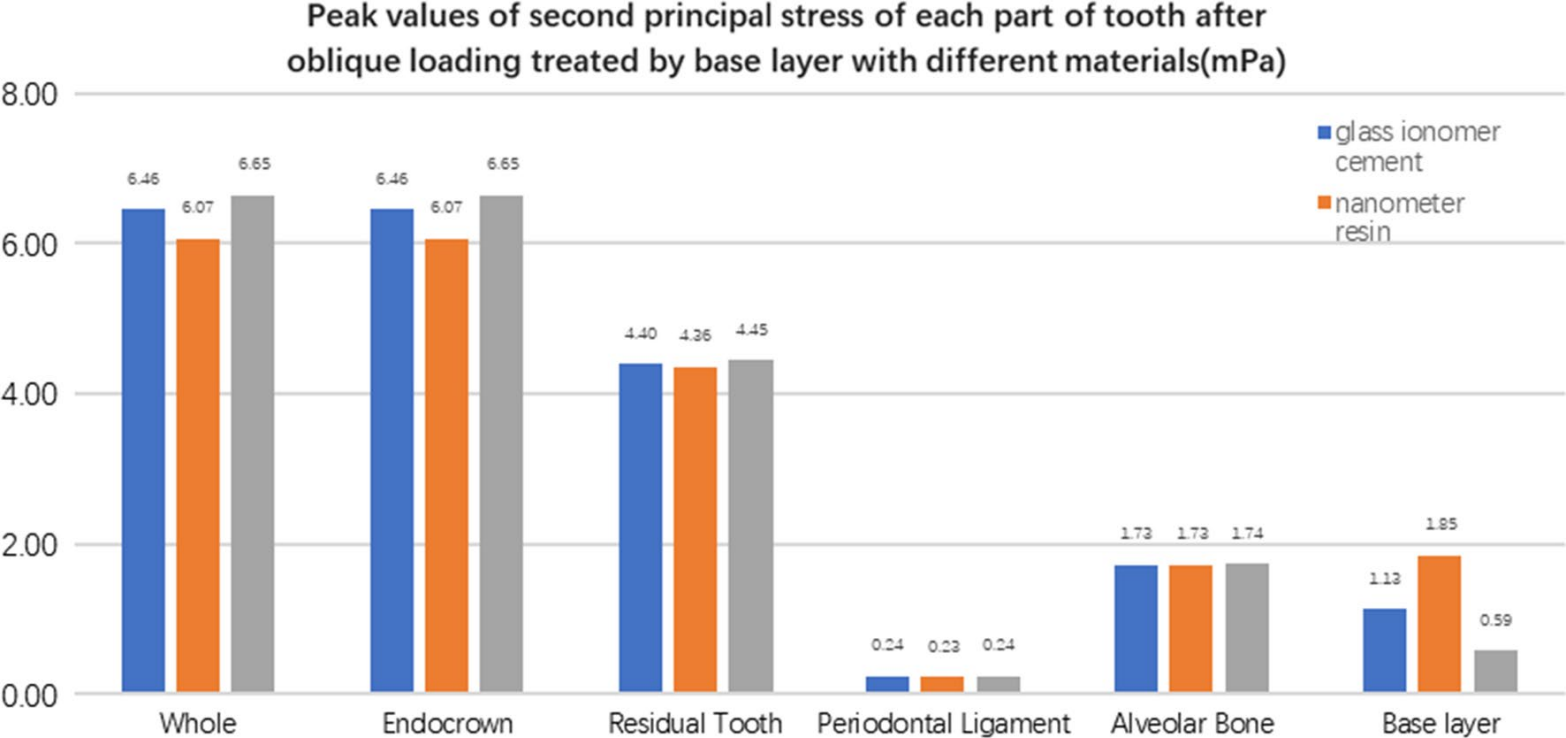
The second stress peak (mPa) of each part of the tooth body treated with different bedding materials under oblique loading

**Fig. 34:**
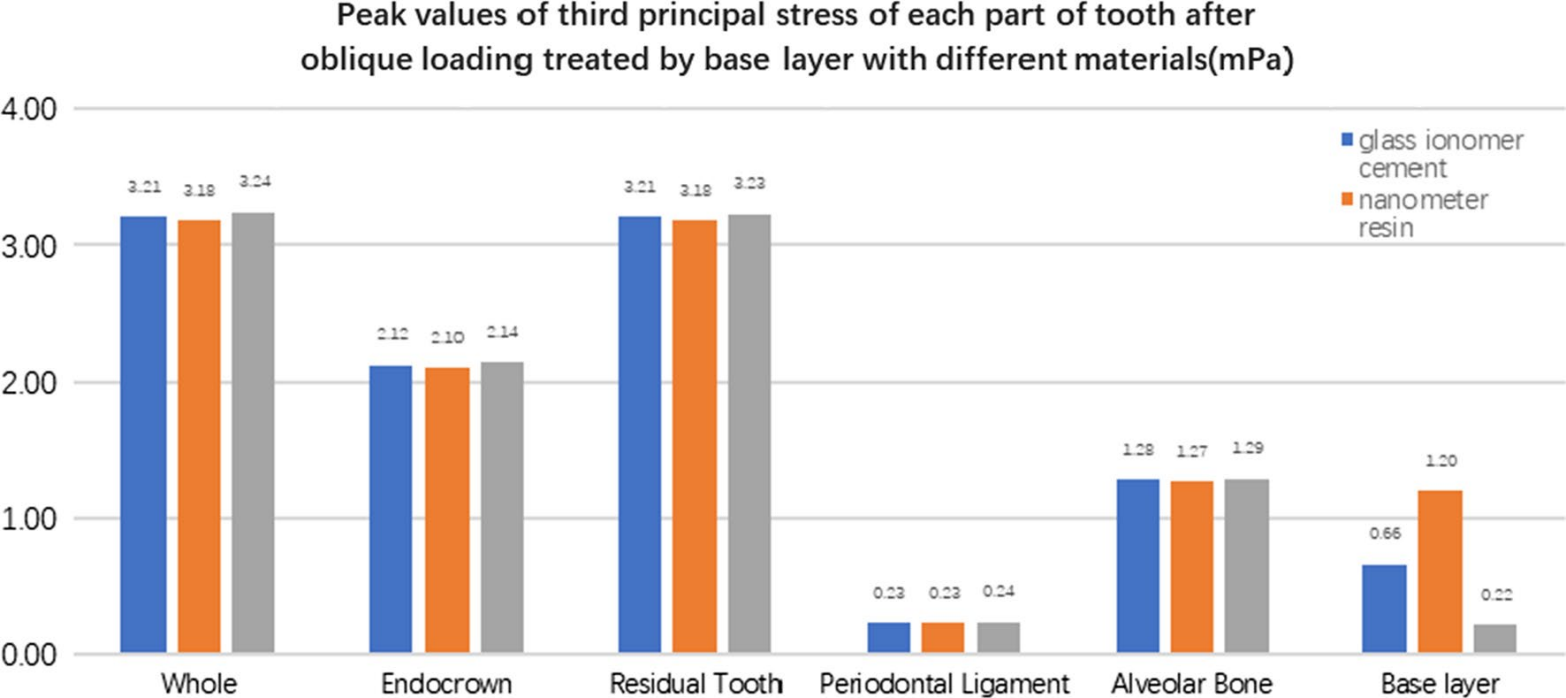
The third stress peak (mPa) of each part of the tooth body treated with different bedding materials under oblique loading

The equivalent stress, first principal stress (σ_1_), second principal stress (σ_2_), and third principal stress (σ_3_) on the restorations, residual teeth, periodontal ligament, and alveolar bone, were essentially the same in the three model groups. The main difference lies in the base layer. Under the two loading conditions, the observation shows that the fluid resin as the base material suffered the smallest stress, and the composite resin was relatively large.

#### *Comparison of strain change trends of different base materials under stress (Fig. **35**, Table **3**)*

**Fig. 35:**
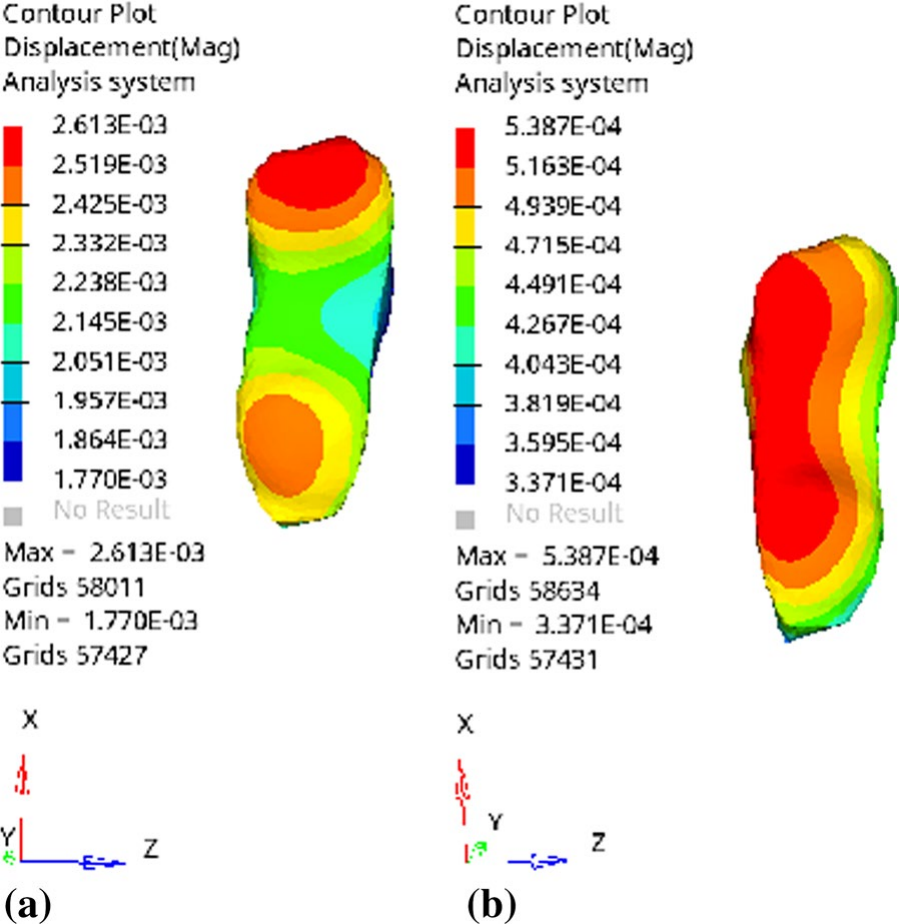
Distribution of strain when the cushion layer material is fluid resin under compression: a Vertical loading; b Oblique loading

**Table 3:** peaks of strain experienced by different base materials

	Glass ion	Fluid resin	Composite resin
Vertical loading	5.300E − 04	5.387E − 04	5.235E − 04
Oblique loading	2.610E − 03	2.613E − 03	2.608E − 03

After the three groups of models were vertically pressurized, the strain of the base layer with fluid resin was relatively large, while that of the composite resin was small. After the models were obliquely pressurized, the strain trends were the same. Taking the fluid resin group model as an example, the vertical compression indicated that the strain peak was located in the region corresponding to the place where the prosthesis was bonded near the center, and the strain peak was located in the corresponding region below the buccal apex under the oblique compression.

## Discussion

### Application of all-ceramic endocrown repair

Bindl and Mormann [4] proposed "endocrown," the medullary cavity retention crown. Through the medullary cavity as the retention, the part that extends into the medullary cavity and covers the surface constitutes the prosthesis. The endocrown and the abutment are annularly butted, and sufficient retention force is obtained by utilizing the embedding force between the axial walls of the abutment on the restoration and the adhesive force provided by the resin adhesive. At present, the main material of endocrown in clinical practice is lithium disilicate glass-ceramic. Grestigt et al. [5] found that the flexural strength of lithium disilicate glass-ceramics under oblique loading was higher than that of multiphase composite resin material, and there was no significant difference under vertical loading. The ceramic provides sufficient strength for the bite and provides improved bond strength with silane coupling. Therefore, IPS emax ceramic was used in this experiment.

### Advantage of endodontically treated maxillary premolar model restored with endocrowns

The anatomic morphology and location of endodontically treated maxillary premolars are special (e.g., the narrowed neck is the weak area of the tooth, making it the one with the largest crown-to-width ratio in maxillary teeth). In addition, because of its position at the corner of the maxillary central dentition, it is subjected to greater lateral forces from the buccolingual direction, so the results of many studies on anterior teeth and molars are not applicable to premolars. The restoration method used in the endodontically treated tooth is one of many influencing factors [6]. For full crown restoration of maxillary first premolars, more tooth tissues need to be ground out, which further loses the flexural strength of abutment teeth, and post-core restoration also increases the incidence of root fracture [7]. For endodontically treated maxillary premolars, the restoration method using embedded medullary cavity is usually adopted in the clinic, in which the prosthesis is directly bonded to the affected teeth through mechanical retention in the medullary chamber, without grinding out a large number of remaining teeth. It is suitable for restoring affected teeth with large-area defects after root canal treatment [8]. This not only increases the retention effect but also protects the weak neck, with a high success rate of the prosthesis [9]. Therefore, the endocrown was used for the model in this experiment.

The adhesive layer interface is complex, and its thickness is too thin compared with that of other dental structures. In addition, since the current glass-ceramic has a high bonding performance, it can be regarded as the same whole body with the prosthesis, with little effect on the overall stress. Therefore, the adhesive layer model was not separately established in this modeling [10].

### Thickness selection of pulp cavity base materials

Moscovich et al. [11] showed that the stress distribution of the inlay was improved with the appropriate base thickness. When there is no base material at the pulp chamber bottom, the crown stress is concentrated at the edge of the hole bottom because the stress is transmitted directly from the prosthesis to the pulp chamber bottom. After the base material is applied to the pulp chamber bottom, the stress at the hole bottom decreases because the stress peak moves from the hole bottom to the contact surface between the base material and the prosthesis. That is, the stress "breaks" phenomenon occurs, and the stress must be transferred from the base layer to the pulp chamber bottom, so the stress tends to be smaller at the pulp chamber bottom. Similarly, the rest of the dental crown pad layer can also play a role in buffering stress. This conclusion has also been verified in this experiment. In the model without base layer treatment, the overall stress is relatively high, and the maximum stress is concentrated on the remaining teeth. There is also an effect of different fluid resin base thickness on the stress transfer in pulpal cemented crowns. From the comprehensive consideration of stress changes under vertical and oblique loading, the protection effect of residual teeth with the thickness of 1 mm was the best, and the effect was better than that without a base layer and with the base layer thickness of 2 mm and 3 mm. Therefore, in this experiment, it is recommended to control the thickness of the medullary cavity base material to 1 mm.

### Stress analysis of different pulp cavity base materials

Glass ionomer cement, zinc phosphate cement, fluid resins, calcium hydroxide, and composite resins are often commonly used as base materials in clinical practice. In this experiment, three of the most commonly used ones were selected as research subjects. Farah et al. found that the elastic modulus of the base material in the medullary cavity plays a major role in stress distribution. For different materials, the elastic modulus and stress distribution are different due to their differences, thus affecting the filling and repair effect [12].

The experimental results showed no significant difference in the equivalent peak stress of the remaining dentin after loading the endodontically treated premolar teeth under different base materials. All of them were lower than the normal tensile strength of dentin. In the case of experimental loading, no fracture of the dentin occurred. According to the results of this experiment, the base layer material had no significant effect on the equivalent stress and the first, second and third principal stresses of tooth and prosthesis. The reason might be that the endocrown coverage was extensive, and the thickness of the base layer was relatively small compared with that of the prosthesis and the tooth, which only served as a buffer and had little effect on the overall stress. As for the analysis of the base layer's stress and strain peak values, the fluid resin exhibited relatively large strain and relatively small stress. The base layer was mainly in contact with the bottom and sidewall of the medullary cavity. With the low elastic modulus of the base layer, the strain generated during occlusion was relatively large, and the stress at the bottom of the cavity decreased with the decrease of the conductive force on the dentin.

In this series of experiments, we have found that the overall stress of endodontically treated premolars is relatively small when the underlayer with an appropriate thickness (1 mm) is used and restored in the form of medullary cavity retention crown. Different base materials have little effect on the stress of restorations and teeth, so the materials commonly used in clinical practice can be selected at present. Materials with a small elastic modulus (such as fluid resin) can reduce the stress in the medullary cavity and achieve the purpose of protecting the dentin. In contrast, the fluid resin is more convenient to operate, has good fluidity, and can achieve the effect of edge sealing.

## Data Availability

All data generated or analysed during this study are included in this published article.

## Abbreviations

STL: STereoLithography
DICOM: Digital Imaging and Communications in Medicine
FEA: Finite element analysis
